# Rank based cointegration testing for dynamic panels with fixed *T*

**DOI:** 10.1007/s00181-017-1304-8

**Published:** 2017-07-07

**Authors:** Artūras Juodis

**Affiliations:** Department of Economics, Econometrics and Finance, Faculty of Economics and Business, University of Groningen, Nettelbosje 2, 9747 AE Groningen, The Netherlands

**Keywords:** Dynamic panel data, Panel VAR, Cointegration, Fixed *T* consistency

## Abstract

In this paper, we show that the cointegration testing procedure of Binder et al. (Econom Theory 21:795–837, 2005) for Panel Vector Autoregressive model of order 1, PVAR(1) is not valid due to the singularity of the hessian matrix. As an alternative we propose a method of moments based procedure using the rank test of Kleibergen and Paap (J Econom 133:97–126, 2006) for a fixed number of time series observations. The test is shown to be applicable in situations with time-series heteroscedasticity and unbalanced data. The novelty of our approach is that in the construction of the test we exploit the “weakness” of the Anderson and Hsiao (J Econom 18:47–82, 1982) moment conditions. The finite-sample performance of the proposed test statistic is investigated using simulated data. The results indicate that for most scenarios the method has good statistical properties. The proposed test provides little statistical evidence of cointegration in the employment data of Alonso-Borrego and Arellano (J Bus Econ Stat 17:36–49, 1999).

## Introduction

In this paper, we consider the cointegration testing problem for Panel VAR model of order 1 with a fixed time dimension. Up to date the only testing approach in this case is the likelihood ratio test based on the transformed maximum likelihood (TML) estimator of Binder et al. (2005) [hereafter BHP]. However, in the univariate setup it is known that for data with autoregressive parameter close to unity, the likelihood approach does not have a gaussian asymptotic limit, see e.g. Kruiniger (2013). We extend that result to multivariate setting and argue that the cointegration testing procedure of Binder et al. (2005) is not valid due to the singularity of the corresponding expected hessian matrix.

To the best of our knowledge, in the fixed *T* dynamic panel data (DPD) literature no feasible method of moments (or least-squares) alternative to likelihood based cointegration testing procedures is available. The main reason for the absence of method of moments based alternatives is that jacobian matrix of the Anderson and Hsiao (1982) moment conditions is of reduced rank, when process is cointegrated. It is natural to use this information and consider a rank based cointegration test, based on the rank of the jacobian matrix. In this paper, we propose such a test and show that it is applicable in situations with time-series heteroscedasticity and, unlike the likelihood based tests, the new test does not require any numerical optimization. At the same time, this procedure cannot provide inference that is uniform over the parameter space, as the asymptotic distribution of the test depends on the properties of the initial condition.

In the Monte Carlo section of this paper, we investigate the finite sample properties of the proposed procedure. We find that the new testing procedure provides a good size control as well as high power in most of the designs considered. However, in some setups this test lacks power if the data generating process for the initial condition substantially deviates from stationarity.

The paper is structured as follows. In Sect. 2, we briefly present the model, the testing problem at hand and the results for the testing procedure of Binder et al. (2005). Rank-based cointegration testing procedure is formally introduced in Sect. 3. In Sect. 4, we continue with the finite sample performance by means of a Monte Carlo analysis. In Sect. 5, we illustrate the testing procedure using the data of Alonso-Borrego and Arellano (1999). Section 6 concludes.

Here we briefly discuss notation. **Bold** upper-case letters are used to denote the original parameters, i.e. $\{\varvec{\varPhi },\varvec{\varSigma },\varvec{\varPsi }\}$, while the lower-case letters $\{\varvec{\phi },\varvec{\sigma },\varvec{\psi }\}$ denote ${\text {vec}}\,{(\cdot )}$ (${\text {vech}}{(\cdot )}$ for symmetric matrices) of corresponding parameters, in the univariate setup corresponding parameters are denoted by $\{\phi ,\sigma ^{2},\psi ^{2}\}$. We use $\rho (\varvec{A})$ to denote the spectral radius^1^ of a matrix $\varvec{A}\in \mathbb {R}^{n\times n}$. We define $\bar{\varvec{y}}_{i-}\equiv (1/T)\sum _{t=1}^{T} \varvec{y}_{i,t-1}$ and similarly $\bar{\varvec{y}}_{i}\equiv (1/T)\sum _{t=1}^{T} \varvec{y}_{i,t}$. We use $\tilde{\varvec{x}}$ to indicate variables after Within Group transformation (for example $\tilde{\varvec{y}}_{i,t}\equiv \varvec{y}_{i,t}-\bar{\varvec{y}}_{i}$), while $\ddot{\varvec{x}}$ are used for variables after a “quasi-averaging” transformation.^2^ For further details, see Abadir and Magnus (2002). Where necessary, we use the 0 subscript to denote the true value of the parameters, e.g. $\varvec{\varPhi }_{0}$.

## Cointegration testing for fixed *T* panels

### The model

In this paper, we consider the following PVAR(1) specification

1 $$\begin{aligned} \varvec{y}_{i,t}=\varvec{\eta }_{i}+\varvec{\varPhi }{}\varvec{y}_{i,t-1}+\varvec{\varepsilon }_{i,t}, \quad i=1, \ldots , N,\quad t=1, \ldots , T, \end{aligned}$$

where $\varvec{y}_{i,t}$ is an $[m \times 1]$ vector, $\varvec{\varPhi }$ is an $[m \times m]$ matrix of parameters that are of main interest, $\varvec{\eta }_{i}$ is an $[m \times 1]$ vector of unobserved individual specific covariates, and $\varvec{\varepsilon }_{i,t}$ is an $[m \times 1]$ vector of innovations independent across *i*, with zero mean and some finite covariance matrix. If one sets $m=1$, the model reduces to the univariate linear dynamic panel data model with autoregressive dynamics.

Throughout this paper we assume that $\varvec{\eta }_{i}$ satisfy the so-called “common dynamics” assumption

$$\begin{aligned} \varvec{\eta }_{i}=(\varvec{I}_{m}-\varvec{\varPhi })\varvec{\mu }_{i}, \end{aligned}$$

where $\varvec{\varPi }\equiv \varvec{\varPhi }-\varvec{I}_{m}$. If at least one eigenvalue of $\varvec{\varPhi }$ is equal to unity this assumption ensures that there is no discontinuity in the data generating process (DGP), for further discussion, see e.g. BHP.

Assuming common dynamics we can rewrite the model in (1) as

2 $$\begin{aligned} \Delta {}\varvec{y}_{i,t}=\varvec{\varPi }\varvec{u}_{i,t-1}+\varvec{\varepsilon }_{i,t}, \quad i=1, \ldots , N,\quad t=1, \ldots , T. \end{aligned}$$

Here we define $\varvec{u}_{i,t-1}\equiv \varvec{y}_{i,t-1}-\varvec{\mu }_{i}$. We say that series $\varvec{y}_{i,t}$ are *cointegrated* if the $\varvec{\varPi }$ matrix is of reduced rank $0<r<m$.^3^ In particular, there exist full column rank (of rank *r*) matrices $\varvec{\alpha }_{r}$ and $\varvec{\beta }_{r}$ such that:^4^

3 $$\begin{aligned} \varvec{\varPhi }=\varvec{I}_{m}+\varvec{\alpha }_{r}\varvec{\beta }_{r}', \end{aligned}$$

where *r* is the rank of $\varvec{\varPi }$. Matrices $\varvec{\alpha }_{r}$ and $\varvec{\beta }_{r}$ are not unique, as for any $[r\times r]$ invertible matrix $\varvec{U}$

$$\begin{aligned} \varvec{\alpha }_{r}\varvec{\beta }_{r}'=\varvec{\alpha }_{r}\varvec{U}\varvec{U}^{-1}\varvec{\beta }_{r}'=\varvec{\alpha }_{r}^{*}\varvec{\beta }_{r}^{*'}. \end{aligned}$$

This is the so-called “rotation problem”. As a result, it is a usual practice in the literature to introduce identifying restrictions on $\varvec{\alpha }_{r}$ or $\varvec{\beta }_{r}$. To construct the test statistic that we formally introduce in Sect. 3.1, we follow common practise and assume that $\varvec{\beta }_{r}'=(\varvec{I}_{r},\varvec{\varDelta })$, where $\varvec{\varDelta }$ is an $[r\times m-r]$ matrix.

As a natural starting point one can consider the fixed effects (FE) estimator for $\varvec{\varPhi }$ parameter

4 $$\begin{aligned} \hat{\varvec{\varPhi }}'=\left( \sum _{i=1}^{N}\sum _{t=1}^{T}\tilde{\varvec{y}}_{i,t-1}\tilde{\varvec{y}}_{i,t-1}'\right) ^{-1} \sum _{i=1}^{N}\sum _{t=1}^{T}\tilde{\varvec{y}}_{i,t-1}\tilde{\varvec{y}}_{i,t}'. \end{aligned}$$

It is well known that the estimator of this type is inconsistent for fixed *T*, see e.g. Nickell (1981) and Hahn and Kuersteiner (2002). For the unit root case, i.e. $\varvec{\varPhi }_{0}=\varvec{I}_{m}$ (correspondingly $r=0$), one can show that

5 $$\begin{aligned} \mathop {{{\mathrm{plim}}}}\limits _{N \rightarrow \infty }\hat{\varvec{\varPhi }}=\left( 1-\frac{3}{(T+1)}\right) \varvec{I}_{m}, \end{aligned}$$

suggesting that for $T>2$ we can obtain a consistent estimate of $\varvec{\varPhi }_{0}=\varvec{I}_{m}$, by considering the bias corrected estimator of the form

6 $$\begin{aligned} \hat{\varvec{\varPhi }}_{BC}\equiv \frac{T+1}{T-2}\hat{\varvec{\varPhi }}. \end{aligned}$$

The bias corrected estimator $\hat{\varvec{\varPhi }}_{BC}$ can be used for unit root testing, see e.g. Harris and Tzavalis (1999) and Kruiniger and Tzavalis (2002) for the univariate case. However, no comparable result is available when series are cointegrated, i.e. $0<r<m$. Alternatively, one can rely on the bias-corrected approaches of Bun and Carree (2005), Ramalho (2005) or Dhaene and Jochmans (2016) to obtain a consistent estimator of $\varvec{\varPhi }$. However, these procedures generally fail to guarantee correct asymptotic inference for reasons similar to those discussed in the next section.^5^

### The likelihood ratio test of BHP

In this section we introduce likelihood based testing and estimation approach advocated by BHP. First, we list the assumptions (that we abbreviate as, TML) used to derive asymptotic distribution of the transformed maximum likelihood estimator:

1. The error terms $\varvec{\varepsilon }_{i,t}$ are i.i.d. across *i* and uncorrelated over time ${\text {E}}[\varvec{\varepsilon }_{i,t}\varvec{\varepsilon }_{i,s}']=\mathbf {O}_{m}$ for $s\ne t$, and ${\text {E}}[\varvec{\varepsilon }_{i,t}\varvec{\varepsilon }_{i,t}']=\varvec{\varSigma }$ for $t>0$. ${\text {E}}[\Vert \varvec{\varepsilon }_{i,t}\Vert ^{4}]<\infty$ holds $\forall t$.
2. The initial deviations $\varvec{u}_{i,0}\equiv \varvec{y}_{i,0}-\varvec{\mu }_{i}$ are i.i.d. across *i*, with ${\text {E}}[\varvec{u}_{i,0}]=\varvec{0}_{m}$ and positive definite ${\text {E}}[\varvec{u}_{i,0}\varvec{u}_{i,0}']=\varvec{\varSigma }_{\varvec{u}_{0}}$. ${\text {E}}[\Vert \varvec{u}_{i,0}\Vert ^{4}]<\infty$ holds.
3. The following moment restrictions are satisfied: ${\text {E}}[\varvec{\varPi }\varvec{u}_{i,0}\varvec{\varepsilon }_{i,t}']=\mathbf {O}_{m}$ for all *i* and $t=1,\ldots ,T$.
4. $$N \rightarrow \infty $$, *T* is fixed.
5. Denote by $\varvec{\kappa }$ a $[k \times 1]$ vector of unknown coefficients. $\varvec{\kappa }\in \varvec{\varGamma }$, where $\varvec{\varGamma }$ is a compact subset of $\mathbb {R}^{k}$ and $\varvec{\kappa }_{0}\in \mathrm {interior}(\varvec{\varGamma })$, while $\rho (\varvec{\varPhi }_{0})\le 1$.

Assumption (TML 1) is the no serial correlation assumption. Note how Assumption (TML 2) places moment restrictions only on one linear combination of $\varvec{y}_{i,0}$ and $\varvec{\mu }_{i}$, rather than separately on $\varvec{y}_{i,0}$ and $\varvec{\mu }_{i}$. Assumption (TML 3) imposes zero covariance restriction on initial deviation and the error terms, which is a standard assumption in the literature. For $\varvec{\varPi }=\mathbf {O}$, ${\text {E}}[\varvec{u}_{i,0}\varvec{\varepsilon }_{i,t}']$ is unrestricted, as $\Delta \varvec{y}_{i,1}$ is not a function of $\varvec{u}_{i,0}$. Assumption (TML 5) is the main exception as compared to the setup in e.g. Juodis (2016), as in this paper we allow the maximum eigenvalue of the autoregressive matrix $\varvec{\varPhi }_{0}$ to be 1. The exact components and dimension *k* of the $\varvec{\kappa }$ vector are related to a particular parametrization of the parameter space used for estimation. Assumptions TML are almost identical to the corresponding assumption FE in Binder et al. (2005), the only difference is that some of their assumptions are “high-level” (e.g. bounds on covariances), while we consider “low-level” assumptions by imposing restrictions directly on $\varvec{\varepsilon }_{i,t}$ and $\varvec{u}_{i,0}$.

The quasi log-likelihood function for $\Delta \varvec{Y}_{i}={\text {vec}}\,{(\Delta {}\varvec{y}_{i,1},\ldots ,\Delta {}\varvec{y}_{i,T})}$ is then defined as follows (up to a constant):

7 $$\begin{aligned} \ell (\varvec{\kappa })\equiv -\frac{N}{2}\log {|\varvec{\varSigma }_{\Delta {}\varvec{\tau }}|}-\frac{N}{2}{\text {tr}}{\left( \left( \varvec{R}'\varvec{\varSigma }_{\Delta {}\varvec{\tau }}^{-1}\varvec{R}\right) \frac{1}{N}\sum _{i=1}^{N}\Delta {}\varvec{Y}_{i}\Delta {}\varvec{Y}_{i}'\right) }, \end{aligned}$$

where $\varvec{\kappa }=(\varvec{\phi }',\varvec{\sigma }',\varvec{\psi }')'$, hence $k=m^{2}+m(m+1)$ and $\varvec{\varPsi }$ is the variance-covariance matrix of the initial observation $\Delta {}\varvec{y}_{i,1}$. The $\varvec{\varSigma }_{\Delta \varvec{\tau }}$ matrix has a block tri-diagonal structure, with $-\varvec{\varSigma }$ on first lower and upper off-diagonal blocks, and $2\varvec{\varSigma }$ on all but the first (1, 1) diagonal blocks. The first (1,1) block is set to $\varvec{\varPsi }$ which takes into account the fact that we do not restrict $\Delta {}\varvec{y}_{i,1}$ to be covariance stationary.^6^ The $[mT \times mT]$ $\varvec{R}$ matrix has $\varvec{I}_{m}$ matrices on the diagonal blocks, and $-\varvec{\varPhi }$ on the first lower off-diagonal blocks.

In Juodis (2016) it is shown that the log-likelihood function of BHP can be substantially simplified to

8 $$\begin{aligned} \ell (\varvec{\kappa })=&-\frac{N}{2}\left( (T-1)\log {|\varvec{\varSigma }|} +{\text {tr}}{\left( \varvec{\varSigma }^{-1}\frac{1}{N}\sum _{i=1}^{N}\sum _{t=1}^{T}(\tilde{\varvec{y}}_{i,t}-\varvec{\varPhi }\tilde{\varvec{y}}_{i,t-1})(\tilde{\varvec{y}}_{i,t}-\varvec{\varPhi }\tilde{\varvec{y}}_{i,t-1})'\right) }\right) \nonumber \\&-\frac{N}{2}\left( \log {|\varvec{\varTheta }|}+{\text {tr}}{\left( \varvec{\varTheta }^{-1}\frac{T}{N}\sum _{i=1}^{N}(\ddot{\varvec{y}}_{i}-\varvec{\varPhi }\ddot{\varvec{y}}_{i-})(\ddot{\varvec{y}}_{i}-\varvec{\varPhi }\ddot{\varvec{y}}_{i-})'\right) }\right) , \end{aligned}$$

where $$\varvec{\kappa }=\left( \varvec{\phi }',\varvec{\sigma }',\varvec{\theta }'\right) '$$ and $$\varvec{\varTheta }\equiv T(\varvec{\varPsi }-\varvec{\varSigma })+\varvec{\varSigma }$$.

If the matrix $\varvec{\varPhi }-\varvec{I}_{m}=\varvec{\alpha }_{r}\varvec{\beta }_{r}'$ is of a reduced rank *r* (cointegration),^7^ this information can be taken into account in estimation and used for testing. To avoid rotational indeterminacy, one can use the same parametrization as BHP and set $\varvec{\beta }_{r}'=\varvec{\delta }_{r}'\varvec{H}_{r}'+\varvec{b}_{r}'$, where $\varvec{H}_{r}$ and $\varvec{b}_{r}$ are known and $\varvec{\delta }_{r}$ is an $[m-r \times r]$ (given $0<r<m$) matrix of parameters. The parameter set in this case is defined as $\varvec{\kappa }_{r} = (({\text {vec}}\,{\varvec{\alpha }_{r}})',({\text {vec}}\,{\varvec{\delta }_{r}'})',\varvec{\sigma }',\varvec{\theta }')'$. Binder et al. (2005) suggest to use the likelihood function (8) in constructing the likelihood ratio test statistic to consistently estimate rank *r*. In particular, the likelihood ratio test statistics for the null hypothesis $H_{0}: r_{0}=r$ against the alternative $H_{A}: r_{A}=r+1$ is of the form

9 $$\begin{aligned} LR(r,r+1)=-2(\ell (\varvec{\kappa }_{r})-\ell (\varvec{\kappa }_{r+1})). \end{aligned}$$

Similarly to the classical maximum eigenvalue test of Johansen (1991) for $N=1$, the overall testing procedure can be performed sequentially, i.e. by first considering $r_{0}=0$ ($\varvec{\varPi }_{0}=\mathbf {O}_{m}$), and if rejected, proceeding with $r_{0}=1$, and so on. BHP argue that this procedure under $H_{0}: r_{0}=r$ has a $\chi ^{2}(\cdot )$ asymptotic limit. In particular, in Remark 4.1. they note that: “Unlike in time-series models, first differencing in panels with *T* fixed still allows identification and estimation of the long-run (level) relations that are of economic interest, irrespective of the unit root and cointegrating properties of the $\varvec{y}_{i,t}$ process.”. As we show next, this conclusion is not completely correct.

One of the standard regularity conditions for extremum estimators, is that the asymptotic (or expected) hessian matrix, ${\varvec{\mathcal {H}}}_{\ell }\equiv {\text {E}}[\varvec{\mathcal {H}}^{N}(\varvec{\kappa }_{0})]$ is positive definite. In Bond et al. (2005), authors showed that for the TML estimator of Hsiao et al. (2002) (which is a special case of Binder et al. 2005 for $m=1$) this regularity condition is violated. In the next theorem we show that the same conclusion extends to a more general case with $m\ge 1$.

#### Theorem 1

(Singularity) Let Assumptions **TML** be satisfied. Then at $\varvec{\varPhi }_{0}=\varvec{I}_{m}$ the ${\varvec{\mathcal {H}}}_{\ell }$ matrix is singular, i.e.

10 $$\begin{aligned} |{\varvec{\mathcal {H}}}_{\ell }|=0. \end{aligned}$$

#### Proof

In the Appendix. $\square$

As the TML estimator can be seen as a non-linear MM estimator with the score vector defining the moment conditions, singularity of the ${\varvec{\mathcal {H}}}_{\ell }$ matrix can be seen as a “weak instrument” problem (using the GMM notation). The singularity result in Theorem 1 is of special interest when the inference regarding the rank of $\varvec{I}_{m}-\varvec{\varPhi }_{0}$ is concerned.

It is important to note that despite singularity of ${\varvec{\mathcal {H}}}_{\ell }$, the TML estimator $\hat{\varvec{\kappa }}_{TMLE}$ remains consistent, hence the identification part of Remark 4.1. in BHP is correct. However, as a result of singularity the limiting distribution for this estimator is non-standard. Using the approach of Roznitzky et al. (2000), Ahn and Thomas (2006) showed that in the univariate model (i.e. $m=1$), the TML estimator of $\phi$ converges at the $N^{1/4}$ rate to a non-standard distribution.^8^ Additionally, they show that LR test statistic for $H_{0}: \phi _{0}=1$ has a mixture distribution, of a $\chi ^{2}(1)$ random variable and a degenerate random variable that takes value 0 with probability 1, with equal mixing weights of 0.5. In this paper, we do not attempt to study the distributional consequences of the singularity for the LR test and leave it for future research.^9^ Based on results in Dovonon and Renault (2009) (for GMM), it is known that for general rank deficiencies the maximal rate of convergence is $N^{1/4}$. However, no results regarding the behavior of the estimator (see discussion in Dovonon and Hall 2016) and the LR ratio test in cases like ours are available. As a result, it is not obvious that using the critical values from the $\chi ^{2}$ distribution with $(m-r)^{2}$ degrees of freedom results in a conservative test.

Although the unit root model is not of prime importance for the main topic of this paper, Theorem 1 provides a natural starting point for intuition of the next result. For the unit root case (i.e. $\varvec{\varPhi }_{0}=\varvec{I}_{m}$) the expression for ${\varvec{\mathcal {H}}}_{\ell }$ simplifies dramatically as $\varvec{\varSigma }_{0}=\varvec{\varTheta }_{0}$. That allowed us in Theorem 1 to show that $|{\varvec{\mathcal {H}}}_{\ell }|=0$ for any value of $\varvec{\varSigma }_{0}$ and *T*. Unfortunately, no result of this type is available when $\varvec{\varPi }$ is of reduced rank $r>0$. However, some special results can be derived for $T=2$.

#### Proposition 1

Let $\varvec{\varPhi }_{0}$ be such that ${\text {rk}}\,{\varvec{\varPi }_{0}}=r$ and $T=2$ then

11 $$\begin{aligned} {\text {rk}}\,{{\varvec{\mathcal {H}}}_{\ell }}\le 0.5m(m-1)+r^{2}. \end{aligned}$$

#### Proof

In the Appendix. $\square$

This quantity is smaller than $m^{2}$ for all $m\le 4$ (note that the bivariate PVAR model is analyzed in most empirical studies with limited number of time-series observations). It follows that for cases of most empirical value the expected hessian matrix is singular and the corresponding estimator does not have a normal limiting distribution. Although in this paper we do not prove more general results for $T>2$, we performed numerous numerical evaluations of ${\varvec{\mathcal {H}}}_{\ell }$ for larger values of *T* and different combinations of population matrices in the bivariate setup.^10^ For all setups we found that the expected hessian matrix is singular for $r<m$ and of full rank otherwise. Given these results the unit root and cointegration testing procedure of BHP that is based on asymptotic $\chi ^{2}(\cdot )$ critical values is not asymptotically valid.

#### Remark 1

Alternatively, instead of considering likelihood function for observations in first differences one can consider a correlated random effects likelihood function (conditional on $\varvec{y}_{i,0}$) as in Arellano (2016) and Kruiniger (2013). Although we do not formally consider a possible singularity of the hessian matrix for that estimator, we conjecture that the main conclusions of this paper are also applicable to that approach (Ahn and Thomas 2006; Kruiniger 2013 proved this for $m=1$).

#### Remark 2

Note that the results of this section are derived under assumption that $\varvec{\varPsi }$ is estimated without any restrictions, i.e. as suggested by Binder et al. (2005). If one instead imposes some restrictions on this parameter matrix, e.g. covariance stationary, it is possible that the expected hessian matrix has full rank. For example, Kruiniger (2008) considers univariate results, where he shows that for $\phi _{0}=1$, the TML estimator retains standard asymptotic properties if the stationarity assumption is used in estimation.

## Jacobian based testing

### Regularity conditions

In this section we propose an alternative approach to cointegration testing. To explain the intuition of our approach, consider the following Anderson and Hsiao (1982) moment conditions for Panel VAR(1) model (see e.g. BHP)

12 $$\begin{aligned} {\text {vec}}\,{\left( {\text {E}}[(\Delta \varvec{y}_{i,t}-\varvec{\varPhi }\Delta \varvec{y}_{i,t-1})\varvec{y}_{i,t-2}']\right) }=\varvec{0}_{m^{2}},\quad t=2,\ldots ,T, \end{aligned}$$

where only the most recent lag, $\varvec{y}_{i,t-2}$ is used as an instrument (other choices are discussed later in the paper). The (minus) jacobian of these moment conditions is given by

13 $$\begin{aligned} \left( {\text {E}}[\Delta \varvec{y}_{i,t-1}\varvec{y}_{i,t-2}']\right) '\otimes \varvec{I}_{m},\quad t=2,\ldots ,T. \end{aligned}$$

From the properties of the Kronecker product it follows that the rank of this matrix is determined by the rank of the matrix inside the brackets.^11^ That term can be expanded as follows (upon redefining $t\rightarrow t+1$, as the previous expression is well defined for $t-1=T$)

14 $$\begin{aligned} {\text {E}}[\Delta {}\varvec{y}_{i,t}\varvec{y}_{i,t-1}']=\varvec{\varPi }{\text {E}}[\varvec{u}_{i,t-1}\varvec{y}_{i,t-1}']+{\text {E}}[\varvec{\varepsilon }_{i,t}\varvec{y}_{i,t-1}']. \end{aligned}$$

Under the no serial correlation assumption, e.g. (TML 1), ${\text {E}}[\varvec{\varepsilon }_{i,t}\varvec{y}_{i,t-1}']=\mathbf {O}_{m}$, while the first term is the product of rank *r* and rank $r_{uy}\le m$ matrices. As a result ${\text {rk}}\,{({\text {E}}[\Delta {}\varvec{y}_{i,t}\varvec{y}_{i,t-1}'])}=\min {(r,r_{uy})}<m$ and this leads to a violation of the “relevance” condition for the Instrumental Variable (IV) estimator, thus the Anderson and Hsiao (1982) moment conditions cannot be used to consistently estimate $\varvec{\varPhi }$.^12^ However, we can use the jacobian matrix directly to test for cointegration, avoiding the estimation step.

Next, we list assumptions that are sufficient to derive the asymptotic properties of the testing approach that we introduce in this section. For the purpose of this section we deviate from (TML) assumptions and restrict moments of $\varvec{\mu }_{i}$ and $\varvec{y}_{i,0}$ separately, rather than their linear combination.

1. The error terms $\varvec{\varepsilon }_{i,t}$ are i.i.d. across *i* and uncorrelated over time, ${\text {E}}[\varvec{\varepsilon }_{i,t}\varvec{\varepsilon }_{i,s}']=\mathbf {O}_{m}$ for $s\ne t$, and ${\text {E}}[\varvec{\varepsilon }_{i,t}\varvec{\varepsilon }_{i,t}']=\varvec{\varSigma }_{t}$ for $t>0$. ${\text {E}}[\Vert \varvec{\varepsilon }_{i,t}\Vert ^{4}]<\infty$ holds $\forall t$.
2. The $\varvec{\mu }_{i}$ are i.i.d. across *i*, with ${\text {E}}[\varvec{\mu }_{i}]=\varvec{0}_{m}$ and ${\text {E}}[\varvec{\mu }_{i}\varvec{\mu }_{i}']=\varvec{\varSigma }_{\varvec{\mu }}$. Furthermore, for all *i* and $t\ge 0$, ${\text {E}}[\varvec{\mu }_{i}\varvec{\varepsilon }_{i,t}']=\mathbf {O}_{m}$. ${\text {E}}[\Vert \varvec{\mu }_{i}\Vert ^{4}]<\infty$ holds.

Note that we allow $\varvec{\varepsilon }_{i,t}$ to be heteroscedastic over time. However, cross-sectional heteroscedasticity is in general not allowed, as in this case ${\text {E}}[\Delta {}\varvec{y}_{i,t}\varvec{y}_{i,t-1}']$ is individual specific, and we cannot consistently estimate both the mean and the variance of $\Delta {}\varvec{y}_{i,t}\varvec{y}_{i,t-1}'$. In particular, for this reason we assume that $\varvec{\mu }_{i}$ are iid. As we consider fixed *T* panels, it is important to explicitly specify DGP for the initial conditions. For this, we assume that $\varvec{y}_{i,0}$ are of the following form

15 $$\begin{aligned} \varvec{y}_{i,0}=\varvec{\varUpsilon }\varvec{\mu }_{i}+\varvec{\varepsilon }_{i,0}. \end{aligned}$$

Here $\varvec{\varUpsilon }$ is an $[m\times m]$ matrix that controls the degree of non-stationarity in the initial condition, i.e. if $\varvec{\varUpsilon }\ne \varvec{I}_{m}$, initial condition is effect non-stationarity.^13^ Note that if $\varvec{\varUpsilon }=\varvec{I}_{m}$ the assumption (*A*.2) can be somewhat relaxed allowing heterogenous $\varvec{\mu }_{i}$. What is left is to specify assumptions on $\varvec{\varepsilon }_{i,0}$. Below we list a few DGPs for $\varvec{\varepsilon }_{i,0}$ that can be used for our purpose.

1. $\varvec{\varepsilon }_{i,0}\sim (\varvec{0}_{m},\varvec{\varSigma }_{0})$ with $\varvec{\varSigma }_{0}$ positive (semi-)definite matrix.
2. $\varvec{\varepsilon }_{i,0}=\sum _{l=0}^{M}\varvec{\varPhi }^{l}\varvec{\varepsilon }_{i,-l}^{*}$. Here *M* is assumed to be finite.
3. $\varvec{\varepsilon }_{i,0}=\sum _{l=0}^{\infty }\left( \varvec{\varPhi }^{l}-\varvec{C}\right) \varvec{\varepsilon }_{i,-l}^{*}+\varvec{C}\varvec{\xi }_{i}$. Here $\varvec{\xi }_{i}$ is an $[m\times 1]$ vector of the (independent) individual-specific initialization effects.^14^

In what follows, we assume that all random variables in (DGP.1)–(DGP.3) satisfy assumptions (A.1)–(A.2). Furthermore, in (DGP.3) for simplicity all $\varvec{\varepsilon }_{i,-l}^{*}$ are homoscedastic over the time-series dimension. This restriction can be relaxed by assuming that all $\varvec{\varSigma }_{-l}^{*}$ are appropriately summable as $l\rightarrow \infty$. (DGP.3) initialization was used in the Monte Carlo studies of BHP and is motivated by the Granger Representation Theorem, see e.g. Theorem 4.2 in Johansen (1995). The (DGP.2), was e.g. used by Hayakawa (2016). It is important to emphasize that all three DGP are well defined for all values of *r*.^15^ ${\text {E}}[\varvec{\varepsilon }_{i,t}\varvec{y}_{i,t-1}']=\mathbf {O}_{m}$ is a direct implication of Assumptions (A.1)–(A.2) and (DGP.1)–(DGP.3).

### Rank test

In this paper, we use the generalized rank test of Kleibergen and Paap (2006) as a basis for our testing procedure. Here we briefly introduce their testing procedure and later apply it to our problem. In construction of the rank test Kleibergen and Paap (2006) use the property that any $[k\times f]$ matrix $\varvec{D}$ can be decomposed as:

$$\begin{aligned} \varvec{D}=\varvec{A}_{q}\varvec{B}_{q}+\varvec{A}_{q,\perp }\varvec{\varLambda }_{q}\varvec{B}_{q,\perp }, \end{aligned}$$

where $\varvec{\varLambda }_{q}$ is a $[(k-q)\times (f-q)]$ matrix and all $\perp$ matrices are defined in the usual way. For $\varvec{\varLambda }_{q}=\mathbf {O}$ the rank of $\varvec{D}$ is determined by the rank of $\varvec{A}_{q}\varvec{B}_{q}$. The procedure in Kleibergen and Paap (2006) is based on testing if $\varvec{\varLambda }_{q}$ is equal to $\mathbf {O}_{(k-q)\times (f-q)}$, with matrices $\varvec{A}_{q},\varvec{B}_{q},\varvec{\varLambda }_{q}$ obtained using the singular value decomposition (SVD). In our case, we consider singular value decomposition of jacobian matrix, i.e. $\varvec{D}={\text {E}}[\Delta \varvec{y}_{i,t}\varvec{y}_{i,t-1}']$.

Next, we define the sample analogue of $\varvec{D}$:

$$\begin{aligned} \overline{\Delta \varvec{y}_{i,t}\varvec{y}_{i,t-1}'}\equiv \frac{1}{N}\sum _{i=1}^{N}\Delta {}\varvec{y}_{i,t}\varvec{y}_{i,t-1}'. \end{aligned}$$

Applying the standard Lindeberg–Levý CLT, it follows that:

$$\begin{aligned} \sqrt{N}{\text {vec}}\,\left( \overline{\Delta \varvec{y}_{i,t}\varvec{y}_{i,t-1}'}-{\text {E}}[\Delta \varvec{y}_{i,t}\varvec{y}_{i,t-1}']\right) \mathop {\longrightarrow }\limits ^{d}, N(\varvec{0}_{m^{2}},\varvec{V}),\quad t=2,\ldots ,T. \end{aligned}$$

Here the full rank matrix $\varvec{V}$ can be consistently estimated using its finite sample counterpart:

$$\begin{aligned} \varvec{V}_{N}=\frac{1}{N}\sum _{i=1}^{N}{\text {vec}}\,{(\Delta {}\varvec{y}_{i,t}\varvec{y}_{i,t-1}')}{\text {vec}}\,{(\Delta {}\varvec{y}_{i,t}\varvec{y}_{i,t-1}')}'-{\text {vec}}\,{\overline{\Delta \varvec{y}_{i,t}\varvec{y}_{i,t-1}'}}{\text {vec}}\,{\overline{\Delta \varvec{y}_{i,t}\varvec{y}_{i,t-1}'}}'. \end{aligned}$$

Consequently, the estimator $\overline{\Delta \varvec{y}_{i,t}\varvec{y}_{i,t-1}'}$ satisfies sufficient conditions in Kleibergen and Paap (2006).^16^ As a result one can apply Theorem **1** of Kleibergen and Paap (2006) to the problem at hand:

#### Theorem 2

Let Assumptions (A.1)–(A.2) be satisfied with $\varvec{\varepsilon }_{i,0}$ generated by one of (DGP.1)–(DGP.3), then:

$$\begin{aligned} \sqrt{N}\hat{\varvec{\lambda }}_{r}\mathop {\longrightarrow }\limits ^{d}N(\varvec{0}_{(m-r)^{2}},\varvec{\varOmega }_{r}), \end{aligned}$$

where

$$\begin{aligned} \hat{\varvec{\lambda }}_{r}&={\text {vec}}\,{\hat{\varvec{\varLambda }}_{r}},\quad \hat{\varvec{\varLambda }}_{r}=\hat{\varvec{A}}_{r,\perp }'\overline{\Delta \varvec{y}_{i,t}\varvec{y}_{i,t-1}'}\hat{\varvec{B}}_{r,\perp }',\\ \varvec{\varOmega }_{r}&=(\varvec{B}_{r,\perp }\otimes \varvec{A}_{r,\perp }')\varvec{V}(\varvec{B}_{r,\perp }\otimes \varvec{A}_{r,\perp }')' \end{aligned}$$

Furthermore, under $H_{0}: {\text {rk}}\,{{\text {E}}[\Delta \varvec{y}_{i,t}\varvec{y}_{i,t-1}']}=r$, the test statistic:

$$\begin{aligned} rk(r)=N\hat{\varvec{\lambda }}_{r}'\varvec{\varOmega }_{r}^{-1}\hat{\varvec{\lambda }}_{r}' \end{aligned}$$

converges in distribution to a $\chi ^{2}(\cdot )$ random variable with $(m-r)^{2}$ degrees of freedom.

Matrices $\varvec{A}$ and $\varvec{B}$ in Theorem 2 are obtained from the SVD of $\overline{\Delta \varvec{y}_{i,t}\varvec{y}_{i,t-1}'}$. An operational version of the *rk*(*r*) test statistic is obtained by replacing the (unknown) matrix $\varvec{\varOmega }_{r}$ with some consistent estimator. An obvious choice for $\hat{\varvec{\varOmega }}_{r}$ is given by:

$$\begin{aligned} \hat{\varvec{\varOmega }}_{r}=(\hat{\varvec{B}}_{r,\perp }\otimes \hat{\varvec{A}}_{r,\perp }')\varvec{V}_{N}(\hat{\varvec{B}}_{r,\perp }\otimes \hat{\varvec{A}}_{r,\perp }')'. \end{aligned}$$

Note that this test statistic uses the unrestricted estimate of ${\text {E}}[\Delta \varvec{y}_{i,t}\varvec{y}_{i,t-1}']$, hence we do not explicitly specify the alternative hypothesis (as it is done for LR test of e.g. Johansen 1991 or Binder et al. 2005). However, as we discuss next, this testing approach has power only towards alternative with $r_{0}>r$, i.e. test rejects if the true rank is larger than the hypothesized one.

The result in Theorem 2 suggests that one can use a sequential testing procedure in order to determine the rank of $E[\Delta \varvec{y}_{i,t}\varvec{y}_{i,t-1}']$. In particular, one can begin with $H_{0}: {\text {rk}}\,{{\text {E}}[\Delta \varvec{y}_{i,t}\varvec{y}_{i,t-1}']}=0$, and if rejected, consider $H_{0}: {\text {rk}}\,{{\text {E}}[\Delta \varvec{y}_{i,t}\varvec{y}_{i,t-1}']}=1$, and so on, until the first non-rejection. The construction of such sequential procedure does not differ from the one suggested in Johansen (1991) or Binder et al. (2005). However, ${\text {E}}[\Delta \varvec{y}_{i,t}\varvec{y}_{i,t-1}']$ is not of the prime interest for us, as we are interested in testing the rank of $\varvec{\varPi }$. This begs the question:

- **Under which conditions one can interpret rejection/non-rejection of the** $\mathbf{r k(r)}$ **test as an evidence regarding the rank of** $\varvec{\varPi }$?

If one rejects the null hypothesis $H_{0}: {\text {rk}}\,{{\text {E}}[\Delta \varvec{y}_{i,t}\varvec{y}_{i,t-1}']}=r$, one can also reject $H_{0}: {\text {rk}}\,{\varvec{\varPi }_{0}}=r$, as the rank of ${\text {rk}}\,{{\text {E}}[\Delta \varvec{y}_{i,t}\varvec{y}_{i,t-1}']}\le {\text {rk}}\,{(\varvec{\varPi })}$. However, our assumptions do not ensure that $\varvec{\varPi }{\text {E}}[\varvec{u}_{i,t-1}\varvec{y}_{i,t-1}']$ has a reduced rank if and only if $\varvec{y}_{i,t}$ are cointegrated (the “if” part was established above). Hence, it still remains to be investigated under which conditions the ${\text {E}}[\Delta {}\varvec{y}_{i,t}\varvec{y}_{i,t-1}']$ term is of reduced rank if and only if $\varvec{\varPi }$ is of reduced rank. Let us investigate this issue more closely by expanding the ${\text {E}}[\varvec{u}_{i,t-1}\varvec{y}_{i,t-1}']$ term (for $t\ge 2$):

$$\begin{aligned} {\text {E}}[\varvec{u}_{i,t-1}\varvec{y}_{i,t-1}']=&{\text {E}}\left[ \left( \varvec{\varPhi }^{t-1}\varvec{u}_{i,0}+\sum _{s=0}^{t-2}\varvec{\varPhi }^{s}\varvec{\varepsilon }_{i,t-s-1}\right) \right. \\&\quad \left. \left( \varvec{\mu }_{i}+\varvec{\varPhi }^{t-1}\varvec{u}_{i,0}+\sum _{s=0}^{t-2}\varvec{\varPhi }^{s}\varvec{\varepsilon }_{i,t-s-1}\right) '\right] \\ =&\underbrace{\varvec{\varPhi }^{t-1}(\varvec{\varUpsilon }-\varvec{I}_{m})\varvec{\varSigma }_{\varvec{\mu }}(\varvec{\varPhi }^{t-1}(\varvec{\varUpsilon }-\varvec{I}_{m}))'}_{p.s.d}+\varvec{\varPhi }^{t-1}(\varvec{\varUpsilon }-\varvec{I}_{m})\varvec{\varSigma }_{\varvec{\mu }}\\&+\underbrace{\sum _{s=0}^{t-2}\varvec{\varPhi }^{s}\varvec{\varSigma }_{t-1-s}\varvec{\varPhi }^{s'}}_{p.d.}+{\text {E}}[\varvec{\varPhi }^{t-1}\varvec{\varepsilon }_{i,0}\varvec{\varepsilon }_{i,0}'(\varvec{\varPhi }^{t-1})'] \end{aligned}$$

In the effect-stationary setup ($\varvec{\varUpsilon }=\varvec{I}_{m}$) all terms involving $\varvec{\varUpsilon }$ are equal to $\mathbf {O}_{m}$. Furthermore, the third term is a p.d. matrix as all $\varvec{\varSigma }_{s}$ matrices are positive definite. Moreover, (DGP.1)–(DGP.3) assumptions are sufficient to conclude that ${\text {E}}[\varvec{\varPhi }^{t-1}\varvec{\varepsilon }_{i,0}\varvec{\varepsilon }_{i,0}'(\varvec{\varPhi }^{t-1})']$ is also at least positive semi-definite (p.s.d.) matrix. Thus we conclude that the “only if” part is also valid for $\varvec{\varUpsilon }=\varvec{I}_{m}$.

Unfortunately, there is a lot of evidence in the DPD literature suggesting that in general this assumption can be too restrictive, see e.g. Arellano (2003) and Roodman (2009). If $\varvec{\varUpsilon }\ne \varvec{I}_{m}$, the first, third and fourth terms are p.s.d. matrices, while it is not immediately clear what happens with the second term. For the procedure to consistently estimate the rank of $\varvec{\varPi }$ (and not only underestimate it), one has to place additional restriction

(IDN) Matrix $\varvec{\varPhi }^{t-1}(\varvec{\varUpsilon }-\varvec{I}_{m})\varvec{\varSigma }_{\varvec{\mu }}$ is such that ${\text {E}}[\varvec{u}_{i,t-1}\varvec{y}_{i,t-1}']$ has a full rank *m*.

Note that restriction is “high-level” as it imposes restrictions not directly on parameters, but instead on their non-linear function. Intuitively, assumption (IDN) is satisfied if $\varvec{\varPhi }^{t-1}(\varvec{\varUpsilon }-\varvec{I}_{m})\varvec{\varSigma }_{\varvec{\mu }}$ is “large”, with eigenvalues sufficiently bounded away from zero. However, it is not a trivial task to identify the parameter space of $\{\varvec{\varPhi },\varvec{\varUpsilon },\varvec{\varSigma }_{\varvec{\mu }}\}$ for the aforementioned condition to be satisfied. One special case is obtained for $\varvec{\varUpsilon }=\varvec{I}_{m}$ (effect stationarity) with other matrices being unrestricted (at least finite). If we can ensure that $\varvec{\varPhi }^{t-1}(\varvec{\varUpsilon }-\varvec{I}_{m})\varvec{\varSigma }_{\varvec{\mu }}$ is such that ${\text {E}}[\varvec{u}_{i,t-1}\varvec{y}_{i,t-1}']$ has full rank *m*, then ${\text {E}}[\varvec{\varPi }\varvec{u}_{i,t-1}\varvec{y}_{i,t-1}']$ has reduced rank *r* if and only if $\varvec{y}_{i,t-1}$ are cointegrated.^17^ In the Monte Carlo section of this paper, we check the adequacy of the proposed procedure by considering different values of $\varvec{\varUpsilon }$ that are mentioned in the literature.

The test statistic in Theorem 2 is based only on one time series observation (in a sense that if $T>2$, then we can construct a test statistic for every value of *t*, but $t=1$). However, it is not the most efficient way of using the time series information provided. Instead, all time series observations can be pooled into one test statistic to test the rank of:^18^

16 $$\begin{aligned} \overline{\Delta {}\varvec{y}_{i,t}\varvec{y}_{i,t-1}'}_{T}=\frac{1}{N}\sum _{i=1}^{N}\frac{1}{T-1}\sum _{t=2}^{T}\Delta {}\varvec{y}_{i,t}\varvec{y}_{i,t-1}'. \end{aligned}$$

For any fixed value of *T*, the $\overline{\Delta {}\varvec{y}_{i,t}\varvec{y}_{i,t-1}'}_{T}$ term satisfies the sufficient conditions for the CLT, so that the results of Theorem 2 can be extended, with $\varvec{V}_{N}$ for this case given by:

17 $$\begin{aligned} \varvec{V}_{N}= & {} \frac{1}{N}\sum _{i=1}^{N}{\text {vec}}\,{\left( \frac{1}{T-1}\sum _{t=2}^{T}\Delta {}\varvec{y}_{i,t}\varvec{y}_{i,t-1}'\right) }{\text {vec}}\,{\left( \frac{1}{T-1}\sum _{t=2}^{T}\Delta {}\varvec{y}_{i,t}\varvec{y}_{i,t-1}'\right) }'\nonumber \\&-{\text {vec}}\,{\overline{\Delta \varvec{y}_{i,t}\varvec{y}_{i,t-1}'}_{T}} {\text {vec}}\,{\overline{\Delta \varvec{y}_{i,t}\varvec{y}_{i,t-1}'}_{T}}'. \end{aligned}$$

In the next section we use “rk-J” to denote the jacobian based cointegration test for $\overline{\Delta {}\varvec{y}_{i,t}\varvec{y}_{i,t-1}'}_{T}$.

Until now we considered only the jacobian of Anderson and Hsiao (1982) moment conditions, however, for $T>2$ further lags $\varvec{y}_{i,t-j}$, can be used. Nevertheless, it is not clear that the use of lags *j* larger than $j>1$ still ensures that, even in the effect stationary case, ${\text {E}}[\overline{\Delta \varvec{y}_{i,t}\varvec{y}_{i,t-j}'}_{T}]$ has reduced rank *r* if and only if ${\text {rk}}\,{\varvec{\varPi }}=r$. Moreover, the power of the test might be substantially affected by the choice of lags, as with any alternative close to the unit circle we encounter the weak instruments problem for any distanced lags. On the other hand, we can expect a better test power to the alternatives with substantially lower $\rho (\varvec{\varPhi })$.

#### Remark 3

If the model contains time effects $\lambda _{t}$, the test statistic needs to be modified using variables in deviations from their cross-sectional averages $\check{\varvec{y}}_{i,t}\equiv \varvec{y}_{i,t}-(1/N)\sum _{i=1}^{N}\varvec{y}_{i,t}$ rather than levels.

#### Remark 4

One important advantage of the proposed test statistic is the additional flexibility while dealing with unbalanced panels. As long as for every individual *i* at least one $\Delta {}\varvec{y}_{i,t}\varvec{y}_{i,t-1}'$ ($t>1$) term is available, the test statistic can be computed. The only difference as compared to the balanced case is that an individual contribution to $\overline{\Delta {}\varvec{y}_{i,t}\varvec{y}_{i,t-1}'}_{T}$ is no longer a simple averages with $T-1$ terms, but has an individual specific number of observations $T_{i}-1$.

#### Remark 5

The testing procedure remains valid if, as suggested by Kleibergen and Paap (2006), instead of $\overline{\Delta \varvec{y}_{i,t}\varvec{y}_{i,t-1}'}_{T}$ we investigate the rank of $\varvec{D}=\varvec{G}_{N}\overline{\Delta \varvec{y}_{i,t}\varvec{y}_{i,t-1}'}_{T}\varvec{F}_{N}$ (for any full rank matrices ${{\mathrm{plim}}}_{N\rightarrow \infty }\varvec{G}_{N}=\varvec{G}$ and ${{\mathrm{plim}}}_{N\rightarrow \infty }\varvec{F}_{N}=\varvec{F}$). One interesting special case is obtained when we set $\varvec{G}_{N}=\varvec{I}_{m}$ and $\varvec{F}_{N}^{-1}=\frac{1}{N}\sum _{i=1}^{N}\frac{1}{T-1}\sum _{t=2}^{T}\varvec{y}_{i,t-1}\varvec{y}_{i,t-1}'$, as in this case we are testing the rank of the pooled OLS estimator $\hat{\varvec{\varPi }}$. Even though the estimator itself is inconsistent (due to the presence of the unobserved heterogeneity), as we show in this paper, it can be used for estimation of ${\text {rk}}\,{\varvec{\varPi }_{0}}$.

### Discussions

In this section we summarize some of the underlying assumptions, and related problems, for the rk-J test.

**Effect non-stationarity** Recall that results in Theorem 2 are written in terms of the rank of $\overline{\Delta {}\varvec{y}_{i,t}\varvec{y}_{i,t-1}'}_{T}$ rather than $\varvec{\varPi }$. This suggests that if one uses this rank test to perform the sequential procedure in testing the rank of $\varvec{\varPi }$ the procedure is “conservative”, i.e. as for some values of $\varvec{\varUpsilon }$, the jacobian can be of a reduced rank, even if $\varvec{\varPi }$ is of full rank. However, the rank of $\overline{\Delta {}\varvec{y}_{i,t}\varvec{y}_{i,t-1}'}_{T}$ can never be larger than the rank of $\varvec{\varPi }$. In such situations, the rk-J procedure controls the size of the test for $\varvec{\varPi }$, i.e. it rejects in at most $\alpha \%$ cases, and it never gets larger than the nominal level, thus this test controls the size uniformly over ($\varvec{\varSigma }, \varvec{\varUpsilon }$). However, for some combinations of the nuisance parameters ($\varvec{\varSigma }, \varvec{\varUpsilon }$), the power of this test does not converge to 1 as $N\rightarrow \infty$ even if ${\text {rk}}\,(\varvec{\varPi })>r_{0}$, and as a result such testing procedure lacks power. Thus, it is difficult to draw general conclusions about the properties of the rk-J test when one does not reject the null hypothesis and it is likely that $\varvec{y}_{i,t}$ process is not effect stationary.

**Common dynamics assumption** Throughout the paper we maintain the common dynamics assumption for $\varvec{\eta }_{i}$. In the univariate case, it is known that if this assumption is satisfied the moment conditions are not relevant at unity, see a more detailed discussion in Bun and Kleibergen (2016). On the other hand, if the common dynamics assumption is violated, it is possible to have a full rank jacobian even for $\varvec{\varPhi }_{0}=\varvec{I}_{m}$, see the aforementioned paper and the discussion in Hayakawa and Nagata (2016). Hence, even if $\varvec{\varPi }$ matrix is of reduced rank $r<m$ the rank of the jacobian matrix can be of full rank *m*, when the common dynamics assumption is violated. In this case the rejection of the null hypothesis of the rk-J test, is not informative about the underlying rank of the $\varvec{\varPi }$.^19^

**Initialization** For initialization we assumed that ${\text {var}}{\varvec{\varepsilon }_{i,0}}$ is well defined irrespective of the time-series properties of the data. In the univariate setting, it is known that if e.g. ${\text {E}}[\lim _{\phi \rightarrow 1}(1-\phi )\varepsilon _{i,0}^{2}]>0$ then the Anderson and Hsiao (1982) moment conditions have a full rank jacobian matrix. Nevertheless, as discussed in Bun and Kleibergen 2016) this does not imply that $\phi$ parameter is identified.^20^ Note that the initializations of this type would imply that the cross-sectional average of $y_{i,t}$ is not well defined for any $t\ge 0$ which is a rather unrealistic assumption to make.

These issues cannot be underestimated in empirical work. However, at the same time we acknowledge that in order to obtain testing procedures that controls size uniformly^21^ one would have to rely on procedures that are numerically challenging, i.e. subset inference using the continuously updated GMM estimator. We should also emphasize that most of the testing procedures for dynamic panel data (especially for persistent data) fail to guarantee uniform inference over the parameter space of autoregressive parameter and/or initialization of the initial condition.^22^

## Monte Carlo simulations

To the best of our knowledge only the BHP study provides results on cointegration analysis for panels with fixed *T*.^23^ Hence, for the main building blocks of the finite-sample studies performed in this paper we take the setups from BHP, but we provide an extended set of scenarios. Only bivariate panels are considered, thus the only null hypothesis we are testing is:

18 $$\begin{aligned} H_{0}{:} {\text {rk}}\,{\varvec{\varPi }}=1 \end{aligned}$$

For simplicity we use (DGP.2) for initialization:

19 $$\begin{aligned} \varvec{y}_{i,0}=\varvec{\varUpsilon }\varvec{\mu }_{i}+\varvec{\varepsilon }_{i,0}, \quad \varvec{\varepsilon }_{i,0}\sim N\left( \varvec{0}_{2},\sum _{j=0}^{M}\varvec{\varPhi }^{j}\varvec{\varSigma }\varvec{\varPhi }^{j'}\right) . \end{aligned}$$

$\varvec{\varepsilon }_{i,t}$ for all *i* are generates as follows

$$\begin{aligned} \varvec{\varepsilon }_{i,t}&\sim N(\varvec{0}_{2},\varvec{\varSigma }),\quad t>0. \end{aligned}$$

We assume that the error terms are normally distributed i.i.d. both across individuals and time with zero mean and variance-covariance matrix $\varvec{\varSigma }$ (to be specified later). We set $M=50$ 24 and the number of replications $B=10000$.

We generate the individual heterogeneity ($\varvec{\mu }_{i}$) using the exactly same procedure as in BHP:

20 $$\begin{aligned} \varvec{\mu }_{i}=\tau \left( \frac{q_{i}-1}{\sqrt{2}}\right) \check{\varvec{\eta }}_{i},\quad q_{i}\sim \chi ^{2}(1),\quad \check{\varvec{\eta }}_{i} \sim \mathrm {N}(\varvec{0}_{2},\varvec{\varSigma }), \end{aligned}$$

where we set ${\text {vech}}{\varvec{\varSigma }}=\left( .05,.03,.05\right) '$. Following BHP, the variance-covariance matrix of $\check{\varvec{\eta }}_{i}$ coincides with the corresponding variance-covariance matrix used in generating $\varvec{\varepsilon }_{i,t}$.

Before summarizing the design parameters for this Monte Carlo study, recall that $\varvec{\varPi }$ can be rewritten as (for $m=2$):

$$\begin{aligned} \varvec{\varPi }=\varvec{\alpha }\varvec{\beta }'+\lambda \varvec{\alpha }_{\perp }\varvec{\beta }_{\perp }' \end{aligned}$$

We set $\lambda =0$ to study the size of the test, while non-zero values of $\lambda$ are used to investigate power. In particular, the following values of $\lambda$ are considered:

21 $$\begin{aligned} \lambda =\{-0.7;-0.3;-0.1;-0.05;-0.01;-0.005;0.0\}. \end{aligned}$$

In order to reduce the dimensionality of the parameter space we assume that vectors $\varvec{\alpha }$ and $\varvec{\beta }$ are of the following structure:

$$\begin{aligned} \varvec{\alpha }=\alpha \varvec{\imath }_{2},\quad \varvec{\beta }'=(1,-0.2), \end{aligned}$$

and $\alpha =\{-0.1;-0.5\}$. Below we summarize main design parameters of this Monte Carlo study.

$$\begin{aligned} N&=\{50;250;500\},\quad T=\{3;5;7\},\quad \tau =\{1;5\}. \end{aligned}$$

As we discussed in Sect. 3.2 in the effect non-stationary case the particular choice of $\{\varvec{\varUpsilon },\varvec{\varSigma }\}$ and $\tau$ might substantially influence the performance of the test statistic. To address these concerns the following five choices of $\varvec{\varUpsilon }$ are considered:^25^

$$\begin{aligned} \varvec{\varUpsilon }=\left\{ 0.5\varvec{I}_{2};\varvec{I}_{2};1.5\varvec{I}_{2};\varvec{I}_{2}-\varvec{\varPhi }^{10};\left( \begin{array}{cc} .85 &{} .15 \\ .00 &{} .85 \\ \end{array} \right) \right\} . \end{aligned}$$

The choice of $\varvec{\varUpsilon }^{(4)}$ is motivated by the finite start-up assumption, so that the individual specific effects are accumulated only over 9 periods. The particular choice of $S=10$ was rather arbitrary and is not empirically or theoretically motivated.^26^

Comparing our setup to BHP, we can see that design 3 of BHP is achieved when $\alpha =-0.5$ and $\lambda =0.0$ (as they consider size). In order to match our designs with the empirical application, we also considered $N=750$, however the results are qualitatively and quantitatively similar to $N=500$, thus omitted. Other design parameters are also chosen to match some of the properties of the empirical application, as $\varvec{\varUpsilon }^{(5)}$ is based on the estimates in Arellano (2016) obtained from the bivariate panel of Spanish firm data.

In terms of the test power, we suspect that it should be decreasing with $|\lambda |$, with almost no power against alternatives with $\lambda \approx 0$. However, it is very likely that for general $\varvec{\varUpsilon }$ matrices the power curve might not be monotonic because $\lambda$ not only controls the rank of $\varvec{\varPi }$ but as well (indirectly) the eigenvalues of the ${\text {E}}[\varvec{u}_{i,t-1}\varvec{y}_{i,t-1}']$ matrix. Hence, for some specific choices of $\varvec{\varUpsilon }$ we can observe the weak instruments problem of Anderson and Hsiao (1982) moment conditions that is not caused by the reduced rank of $\varvec{\varPi }$ matrix.

### Results

The results for all designs are summarized at the top part of Tables 6, 7, and 8 ($\theta =0$). All rejection frequencies are rounded up to two digits. Empty entries indicate maximal power of 1, 00.

**General patterns** First of all, we can observe that rejection frequencies are monotonically decreasing in $|\lambda |$ for the vast majority of designs without spatial dependence. As we discussed in Sect. 3.2 this property should not be taken as granted for the rk-J test (as dependence on $\varvec{\varPhi }$ is non-linear). For lower values of *N* the test tends to be undersized for $T=3$ and oversized for $T=7$.^27^ In the effect stationary case $\tau$ does not play substantial role and only affects the $\varvec{V}$ matrix, but we can still observe that higher value of $\tau$ is associated with slightly lower power. For $N=500$, the rk-J test has notable power even when $\lambda$ is very close to 0. For instance, all rejection frequencies in the effect stationary designs at $\lambda =0.005$ are above 30% and 25% for $\alpha =-0.5$ and $\alpha =-0.1$ respectively. In the vast majority of cases with size distortions being of similar magnitude, the test power for $\alpha = -0.5$ tends to be higher than for $\alpha = -0.1$.

**Effect non-stationarity and non-monotonic power curves** First, we consider rejection frequencies for $\varvec{\varUpsilon }=0.5\times \varvec{I}_{m}$ as this case is most exceptional in terms of observed patterns. In this case we observe power curves that are not monotonic for $\alpha =-0.1$ (especially for $N=250$) and sharply decreasing for $\alpha =-0.5$ if $\tau =5$ and $T=3$. It can be intuitively explained as in this case the effect non-stationarity term in ${\text {E}}[\Delta {}\varvec{y}_{i,t}\varvec{y}_{i,t-1}']$ is negative, driving the whole expression towards the zero matrix (recall the analysis in Hayakawa 2009 for the univariate case). Thus, we have a weak instrument problem under the alternative hypothesis that is not induced by cointegration.^28^ By varying $\lambda$ parameter we directly vary the relative contributions of time invariant and time varying parts of the variance components in ${\text {var}}{\varvec{y}_{i,t}}$. For larger values of $|\lambda |$ the time invariant part is more pronounced, resulting in substantial effects of the “negative” effect stationarity. On the other hand, for $|\lambda |\approx 0$ the idiosyncratic part is dominant and there is no substantial effects of the “negative” effect non-stationary initialization.

#### Remark 6

**Fig. 1:**
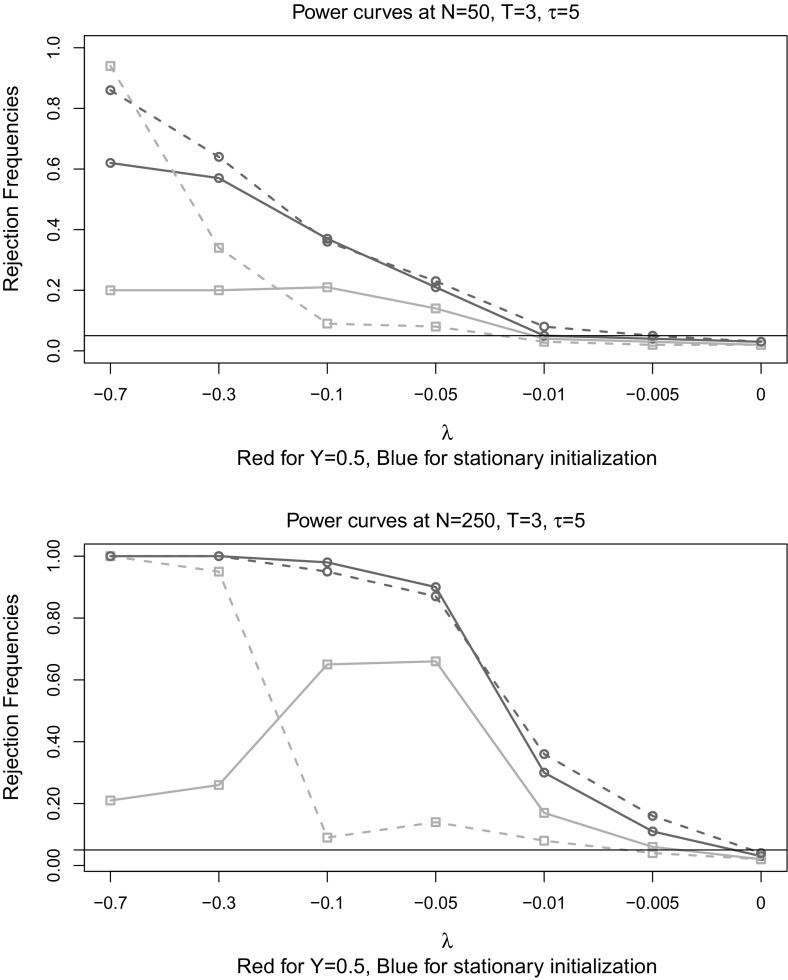
*Red* (*squares*) $\varvec{\varUpsilon }=0.5\varvec{I}_{2}$, *Blue* (*circles*) $\varvec{\varUpsilon }=\varvec{I}_{2}$. *Straight line* $\alpha =-0.1$. *Dashed line* $\alpha =-0.5$. (Color figure online)

This non-monotonicity is further illustrated in Tables 6, 7, where we show how the minimum eigenvalue of the jacobian matrix changes for different nuisance parameters (for very larger *N*). Those patters resemble power curves of the rk-J test as presented in Fig. 1.

As it can be expected, the results for $\varvec{\varUpsilon }=1.5\times \varvec{I}_{m}$ are more straightforward. In this case the power curves are monotonic, and rejection frequencies are uniformly dominating the ones from effect stationary case irrespective of other design parameters. Results for $\varvec{\varUpsilon }^{(4)}$ seem to combine the properties of both $\varvec{\varUpsilon }^{(3)}$ and $\varvec{\varUpsilon }^{(1)}$.^29^ Finally, the results of $\varvec{\varUpsilon }^{(5)}$ are somewhat in between those of $\varvec{\varUpsilon }^{(1)}$ and $\varvec{\varUpsilon }^{(2)}$, but are slightly closer to $\varvec{\varUpsilon }^{(2)}$. It serves as an indication that the off-diagonal element in $\varvec{\varUpsilon }^{(5)}$ is not of any great importance (given the choice of other design parameters).

#### Remark 7

In this paper, we do not provide extensive results for the TML estimator of Binder et al. (2005). The main reason for this (besides theoretical problems discussed in Sect. 2.2) is possibly bimodal log-likelihood function (see e.g. Calzolari and Magazzini 2012; Bun et al. 2016; Juodis 2016). For model with stable dynamics, Juodis (2016) presents several alternatives how one can choose the maximizer of the log-likelihood function from the set of local minimizers. Unfortunately, no results are available for non-stationary dynamics analyzed in this paper. Thus, in order to avoid the situation in which unintentionally test procedure based on the TML estimator performs sub-optimally, we present only some limited results, see Table 5. Results suggest that for alternatives close to the null hypothesis LR test has low power, as the critical value from the $\chi ^{2}(1)$ distribution is too large. On the other hand, for some very distant alternative (where the rk-J test struggles to reject the null hypothesis), LR test has sizeable power.

#### Remark 8

As a robustness check in 6, we also consider model with spatial dependence in the error terms. Evidence of the uniform upward shift in the size can be observed when designs with spatial dependence are considered.

## Empirical illustration

### Data

In this section, we analyze the Spanish firm panel dataset covering 1983–1990 of 738 manufacturing companies from Alonso-Borrego and Arellano (1999). This datasets constitutes a balanced panel of manufacturing companies recorded in the database of the Bank of Spain’s Central Balance Sheet Office from 1983 to 1990. As it contains data only for firms that were observed for the full time span and in all years satisfied specific coherency requirements, it cannot be considered as being a random sample from the population of all firms. For example, this dataset only contains firms that have majority private shareholding, thus state-owned companies are not represented. Thus all results need to be interpreted as conditional on the underlying characteristics used for sample selection.

We construct a bivariate PVAR(1) model with logarithms of employment and wages as dependent variables. Table 1 contains year specific descriptive statistics for these two variables.^30^ Given that cross-sectional means for both variables differ substantially (especially that of wages) between the beginning and the end points, we follow other papers that considered this dataset (e.g. Arellano 2016) and include the time effects in the model, i.e. we consider variables in their deviations from the corresponding year specific cross-sectional averages. The sensitivity to the cross-sectional demeaning is discussed later in this section.

**Table 1:** Descriptive statistics of the dependant variables

Year	$\log \,\,(\mathrm{employment})$				$\log \,\,(\mathrm{wages})$			
	Mean	Median	Min	Max	Mean	Median	Min	Max
1983	4.83	4.82	2.30	9.31	0.45	0.47	$-$0.89	1.32
1984	4.83	4.79	2.30	9.29	0.43	0.45	$-$1.13	1.29
1985	4.83	4.78	2.30	9.21	0.45	0.47	$-$0.91	1.33
1986	4.84	4.79	2.40	9.05	0.52	0.53	$-$0.92	1.55
1987	4.86	4.84	2.40	8.97	0.58	0.59	$-$0.65	1.67
1988	4.88	4.83	2.30	8.91	0.61	0.62	$-$0.77	1.74
1989	4.90	4.86	2.40	8.85	0.67	0.67	$-$0.62	1.83
1990	4.90	4.87	2.30	8.80	0.74	0.75	$-$0.75	1.90

### Results

In contrast to the previous studies that used this data, we investigate the time-series properties of this dataset in a greater detail. In particular, previous studies assumed that GMM and ML estimators are well-behaved, i.e. unit roots and cointegration were excluded a priori. However, estimation results in Arellano (2016) indicate that some estimated parameter values can be close to unit circle, thus non-stationary behaviour cannot be excluded beforehand. In order to elaborate on those observations, we consider a slightly larger set of estimators to obtain point estimates for $\varvec{\varPhi }$ that are valid under different sets of assumption. The results in Table 2 are in line with those in Arellano (2016), with many close to unity point estimates of $\phi _{11}$. The estimates for $\phi _{22}$, on the other hand, are further away from unity, suggesting that both variables can be potentially cointegrated. In order to investigate for possible cointegration in this dataset, we make use of the rk-J procedure that was introduced earlier in this paper.^31^ Specifically, we test if there is a single cointegrating relationship between firms level employment and wages, i.e. $H_{0}: r_{0}=1$.^32^

**Table 2:** Estimation results based on full sample

Estimator	$\phi _{11}$	$\phi _{21}$	$\phi _{12}$	$\phi _{22}$
AB (2)	0.86	$-$0.02	0.14	0.36
AB (1)	0.86	$-$0.03	0.12	0.28
Sys (2)	1.00	0.06	0.07	0.81
Sys (1)	0.99	0.05	0.07	0.81
FE	0.71	0.06	0.08	0.44
FEBC (HK)	0.98	0.02	0.14	0.62
FEBC (K, Sys(2))	1.02	0.02	0.08	0.77
FEBC (SPJ)	1.01	0.02	0.05	0.78
FEBC (BC)	1.05	$-$0.02	0.04	0.74
TMLE $(r=1)$	1.00	0.00	0.07	0.68
TMLE $(r=2)$	1.01	0.01	0.08	0.68

Here “AB$(\cdot )$” and “Sys$(\cdot )$” are the estimators of Arellano and Bond (1991) and Blundell and Bond (1998), respectively. The numbers in brackets indicate, whether these are “two-step” or “one-step” estimates. “FE” denotes the fixed effects estimator. “FEBC” (from top to the bottom) are the bias-correcting fixed effects estimators of Hahn and Kuersteiner (2002), Kiviet (1995) (using “Sys(2)” as the plug-in estimator), Dhaene and Jochmans (2015), Bun and Carree (2005), Juodis (2013). “TMLE” are the Transformed Maximum likelihood estimators with and without rank restrictions imposed on $\varvec{\varPhi }$

First, we apply the rk test of Kleibergen and Paap (2006) directly to GMM estimates $\hat{\varvec{\varPi }}$. We restrict the set of GMM estimators to two step estimators that are also presented in BHP: “AB-GMM” stands for the estimator of Arellano and Bond (1991), while “Sys-GMM” is the estimator of Blundell and Bond (1998) that incorporates moment conditions in levels. Second, the LR tests based on the transformed maximum likelihood function of BHP (LR-TMLE) and maximum likelihood function of Arellano (2016) (as mentioned in Remark 1), (LR-RMLE), are considered. Finally, the “rk-J” test of Sect. 3.2 is considered. Under $H_{0}: {\text {rk}}\,{\varvec{\varPi }_{0}}=1$, if no singularities in the corresponding asymptotic distributions are present, all tests have a $\chi ^{2}(1)$ limit. Note that we present results for “AB-GMM” for informal comparison only, as under $H_{0}: r_{0}=1$ this estimator is not consistent. Results are summarized in Table 3.

**Table 3:** Cointegration testing based on full sample

Name	Test statistic
**AB-GMM**	14.46 (7.20)
Sys-GMM	4.88** (1.31)
LR-TMLE	0.59
LR-RMLE	0.55
rk-J	13.35***

For GMM estimators test statistics based on Windmeijer (2005) corrected 2-step standard errors, are presented in parenthesis. To define significance we use the critical value from the $\chi ^{2}(1)$ distribution. The $5\%$ critical value is 3.84

*$$p < 0.1$$; **$$p < 0.05$$; ***$$p < 0.01$$

From Table 3 we can see that only the rk-J test based on the Anderson and Hsiao (1982) moment conditions rejects $H_{0}$. Results for system GMM estimator are mixed, as based on Windmeijer (2005) corrected standard errors the null hypothesis is not rejected, while it is rejected when using the conventional two-step standard errors. Numerous reasons might account for differences in conclusions. First of all, we suspect that the initialization moment conditions of the System estimator are not valid and it does not come as a surprise that this estimator fails to reject $H_{0}$. Hayakawa and Nagata (2016) provide some evidence based on an incremental Sargan test in support of the latter statement.^33^ Another explanation of results in Table 3 might be the low power of cointegration test used directly on the estimate of $\varvec{\varPi }$.

Now we turn our attention to the likelihood ratio tests. Based on analytical results in this paper for $T=2$ we can suspect that the likelihood procedures under $H_{0}$ of cointegration lack power for close alternatives (recall limited MC results in Table 5), as $\chi ^{2}(1)$ is a poor approximation of the finite sample distribution. Furthermore, we know that both likelihood methods are robust to violations of mean stationarity, but are not so to time-series heteroscedasticity. Thus, we can not rule out the possibility that it can be one of the reasons for divergence in conclusions.^34^

### Sub-sample analysis

In the previous section we investigated the relationships between the firm level employment and wages in the model estimated using the full length of the dataset. Using the rk-J procedure, no significant statistical evidence was found favouring cointegration between the two variables. In this section, we investigate the sensitivity of this conclusion to smaller values of *T* by means of the analysis over sub-samples of the original data. We also check the sensitivity towards inclusion/non-inclusion of the time effects in the model. The results of this section are summarized in Table 4, where in total 6 different sub-samples are considered. First of all, we observe that the non-inclusion of time effects leads to an increase of the test statistic in all sub-samples. The difference is especially pronounced for all subsamples with 1990 as the final year. Overall, irrespective of the time span considered the $rk-J$ statistic rejects the null hypothesis if the time-effects are not included, i.e. data is not cross-sectionally demeaned before estimation.

**Table 4:** Sub-sample $rk-J$ test

Years	*T*	Time effects	
		Yes	No
1983–1990	7	13.35***	29.01***
1983–1989	6	16.09***	35.04***
1983–1988	5	15.60***	28.35***
1983–1987	4	18.74***	20.14***
1984–1990	6	4.59**	27.19***
1985–1990	5	2.57	21.54***
1986–1990	4	0.79	15.94***

*$$ p < 0.1$$; **$$p < 0.05$$;

***$$p < 0.01$$

The same cannot be generally said when data is cross-sectionally demeaned. Note how the value of test statistic increases as *T* increases for sub-sample ending in 1990. In particular, for $T=\{4;5\}$ the null hypothesis is not rejected at any conventional significance level. This behavior emphasizes the value of additional time-series observations and possible lack of power for small values of *T*. As it can be seen from Table 4 the observations for 1983 are especially informative about the properties of the bivariate system, as for all sub-samples starting in 1983 the test statistic always rejects the null hypothesis.

Overall, omission of time-effects from the model does not affect the conclusions from Sect. 5.2. However, a moderate amount of time variation in the magnitude of test statistics suggests that this conclusion is sensitive to different estimation horizons.

## Conclusions

In this paper, we study the properties of the standard Anderson and Hsiao (1982) moment conditions in a PVAR(1) for cointegrated processes. Under the assumptions similar to Binder et al. (2005) we show that these moment conditions are of reduced rank if the process is cointegrated. Based on this observation we propose a rank based test for the null hypothesis of cointegration. We prove that testing procedure in Binder et al. (2005) is invalid due to the singularity of the hessian matrix for persistent data. Monte Carlo results suggest that for most designs, the new test is reasonably sized and has good power properties but might exhibit non-monotonic power curves for models with substantial effect non-stationarity. We apply our testing procedure to the Spanish manufacturing data of Alonso-Borrego and Arellano (1999) and, unlike the test of BHP, we find no evidence of cointegration.

## Appendix

### Proofs: Transformed maximum likelihood

**Notation**

The commutation matrix ${{\mathrm{\mathbf {K}}}}_{a,b}$ is defined such that for any $[a\times b]$ matrix $\varvec{A}$, ${\text {vec}}\,(\varvec{A}')={{\mathrm{\mathbf {K}}}}_{a,b}{\text {vec}}\,(\varvec{A})$. The duplication matrix ${{\mathrm{\mathbf {D}}}}_{m}$ is defined such that for symmetric $[a\times a]$ matrix ${\text {vec}}\,{\varvec{A}}={{\mathrm{\mathbf {D}}}}_{m}{\text {vech}}{\varvec{A}}$.

First, we define a set of new auxiliary variables, which are handy during the derivations of differentials:

#### Results from Binder et al. (2005) and Juodis (2016)

Define

$$\begin{aligned} \varvec{W}_{N}(\varvec{\kappa })\equiv \varvec{\varSigma }^{-1}\sum _{i=1}^{N}\sum _{t=1}^{T}(\tilde{\varvec{y}}_{i,t}-\varvec{\varPhi }{}\tilde{\varvec{y}}_{i,t-1})\tilde{\varvec{y}}_{i,t-1}'+T\varvec{\varTheta }^{-1}\sum _{i=1}^{N}(\ddot{\varvec{y}}_{i} -\varvec{\varPhi }{}\ddot{\varvec{y}}_{i-})\ddot{\varvec{y}}_{i-}', \end{aligned}$$

then the score vector associated with the log-likelihood function (8) is given by:

22 $$\begin{aligned} \nabla (\varvec{\kappa }) = \left( \begin{array}{c} {\text {vec}}\,{\left( \varvec{W}_{N}(\varvec{\kappa })\right) } \\ {{\mathrm{\mathbf {D}}}}_{m}'{\text {vec}}\,{\left( -\frac{N}{2}\left( \varvec{\varSigma }^{-1}((T-1)\varvec{\varSigma }-\varvec{Z}_{N}(\varvec{\kappa }))\varvec{\varSigma }^{-1}\right) \right) } \\ {{\mathrm{\mathbf {D}}}}_{m}'{\text {vec}}\,{\left( -\frac{N}{2}\left( \varvec{\varTheta }^{-1}(\varvec{\varTheta }-\varvec{M}_{N}(\varvec{\kappa }))\varvec{\varTheta }^{-1}\right) \right) } \\ \end{array} \right) . \end{aligned}$$

For $0<r<m$ we have the following result.

##### Corollary 1

Let Assumptions **TML** be satisfied. Then the restricted score vector associated with the log-likelihood function (8) under cointegrating restrictions is given by:

23 $$\begin{aligned} \nabla _{r}(\varvec{\kappa }_{r}) = \left( \begin{array}{c} {\text {vec}}\,{\left( \varvec{W}_{N}(\varvec{\kappa }_{r})\varvec{\beta }_{r}\right) } \\ {\text {vec}}\,{\left( \varvec{\alpha }_{r}'\varvec{W}_{N}(\varvec{\kappa }_{r})\varvec{H}_{r}\right) } \\ {{\mathrm{\mathbf {D}}}}_{m}'{\text {vec}}\,{\left( -\frac{N}{2}\left( \varvec{\varSigma }^{-1}((T-1)\varvec{\varSigma }-\varvec{Z}_{N}(\varvec{\kappa }_{r}))\varvec{\varSigma }^{-1}\right) \right) } \\ {{\mathrm{\mathbf {D}}}}_{m}'{\text {vec}}\,{\left( -\frac{N}{2}\left( \varvec{\varTheta }^{-1}(\varvec{\varTheta }-\varvec{M}_{N}(\varvec{\kappa }_{r}))\varvec{\varTheta }^{-1}\right) \right) } \\ \end{array} \right) . \end{aligned}$$

Proof of this corollary is a a trivial extension of the corresponding result in Juodis (2016) for the unrestricted model. In the special case where $m=2$ and $r=1$, $\varvec{\delta }_{1}$ is a scalar while $\varvec{\alpha }$ is a $[2 \times 1]$ vector implying that the corresponding entries of the $\nabla _{r}(\varvec{\kappa }_{r})$ vector do not have a ${\text {vec}}\,{(\cdot )}$ operator in them.

#### Identification with unit roots

##### Proof of Theorem 1

In order to evaluate the expected hessian matrix, we need to separately calculate the expected value for every term presented in the formula for the second differential at the DGP values $\varvec{\kappa }_{0}$. First of all, we note that ${\text {E}}[(T-1)\varvec{\varSigma }_{0}-\varvec{Z}_{N}(\varvec{\kappa }_{0})]=\mathbf {O}_{m}$ as well as ${\text {E}}[\varvec{\varTheta }_{0}-\varvec{M}_{N}(\varvec{\kappa }_{0})]=\mathbf {O}_{m}$, hence the contribution to the hessian matrix of the first four terms in the expression for  is zero. Following, e.g. Juodis (2013) it can be shown:

24 $$\begin{aligned} {\text {E}}\left[ \varvec{Q}_{N}(\varvec{\kappa }_{0})\right] =-\frac{1}{T}\left( \sum _{l=0}^{T-2}(T-1-l)\varvec{\varPhi }_{0}^{l}\right) \varvec{\varSigma }_{0}. \end{aligned}$$

Due to the fact that exact expressions for other terms are rather messy in the general case, they are derived only for the particular case where $\varvec{\varPhi }_{0}=\varvec{I}_{m}$. Under this assumption and assumptions of Binder et al. (2005), it then follows that $\varvec{\varTheta }_{0}=\varvec{\varSigma }_{0}$. The DGP in this case under restriction of common dynamics, simplifies to:

$$\begin{aligned} \varvec{y}_{i,t} = \varvec{y}_{i,t-1}+\varvec{\varepsilon }_{i,t}= \varvec{y}_{i,0} + \sum _{k=1}^{t}\varvec{\varepsilon }_{i,k}, \quad t \ge 1. \end{aligned}$$

At first we consider expectations of the $\varvec{N}_{N}(\varvec{\kappa }_{0})$ term which we can evaluate based on the general result derived in Lemma A.2 Juodis (2016):

$$\begin{aligned} {\text {E}}[\varvec{N}_{N}(\varvec{\kappa }_{0})']&= (\varvec{I}_{m}-\varvec{\varPhi }_{0}){\text {E}}\left[ \varvec{u}_{i,0}\varvec{u}_{i,0}'\right] (\varvec{I}_{m}-\varvec{\varPhi }_{0})'\varvec{\varXi }' + \frac{1}{T}\varvec{\varSigma }_{0}\varvec{\varXi }'\\&=\frac{1}{T}\varvec{\varSigma }_{0}\varvec{\varXi }'=\frac{(T-1)T}{2T}\varvec{\varSigma }_{0}=\frac{T-1}{2}\varvec{\varSigma }_{0}. \end{aligned}$$

It then similarly follows from the general result in Lemma A.1 Juodis (2016) that:

$$\begin{aligned} {\text {E}}[\varvec{P}_{N}]= & {} {\text {E}}\left[ \frac{T}{N}\sum _{i=1}^{N}\ddot{\varvec{y}}_{i-}\ddot{\varvec{y}}_{i-}'\right] \\= & {} \frac{1}{T}\varvec{\varXi }(\varvec{I}_{m}-\varvec{\varPhi }_{0}){\text {E}}\left[ \varvec{u}_{i,0}\varvec{u}_{i,0}'\right] (\varvec{I}_{m}-\varvec{\varPhi }_{0})'\varvec{\varXi }'\\&+\frac{1}{T}\sum _{t=0}^{T-2} \left( \sum _{j=0}^{t}\varvec{\varPhi }_{0}^{j}\right) \varvec{\varSigma }_{0} \left( \sum _{j=0}^{t}\varvec{\varPhi }_{0}^{j}\right) '\\= & {} \frac{1}{T}\sum _{t=0}^{T-2}(t+1)^{2}\varvec{\varSigma }_{0}=\frac{(T-1)(2T-1)}{6}\varvec{\varSigma }_{0}. \end{aligned}$$

What is left is to evaluate the term involving $\varvec{R}_{N}$:

$$\begin{aligned} {\text {E}}[\varvec{R}_{N}] =&{\text {E}}\left[ \sum _{t=0}^{T-1}\varvec{y}_{i,t}\varvec{y}_{i,t}'-T\bar{\varvec{y}}_{i-}\bar{\varvec{y}}_{i-}'\right] \\ =&{\text {E}}\left[ \sum _{t=1}^{T-1}\left( \sum _{k=1}^{t}\varvec{\varepsilon }_{i,k}\right) \left( \sum _{k=1}^{t}\varvec{\varepsilon }_{i,k}\right) '\right. \\&\quad \left. -\frac{1}{T}\left( \sum _{k=0}^{T-2}(T-1-k)\varvec{\varepsilon }_{i,1+k}\right) \left( \sum _{k=0}^{T-2}(T-1-k)\varvec{\varepsilon }_{i,1+k}\right) '\right] \\ =&{\text {E}}\left[ \sum _{t=1}^{T-1}\left( \sum _{k=1}^{t}\varvec{\varepsilon }_{i,k}\varvec{\varepsilon }_{i,k}'\right) -\frac{1}{T}\left( \sum _{k=0}^{T-2}(T-1-k)^{2}\varvec{\varepsilon }_{i,1+k}\varvec{\varepsilon }_{i,1+k}'\right) \right] \\ =&\left( \sum _{t=1}^{T-1}\left( \sum _{k=1}^{t}1\right) -\frac{1}{T}\left( \sum _{k=0}^{T-2}(T-1-k)^{2}\right) \right) \varvec{\varSigma }_{0}\\ =&\left( \sum _{t=1}^{T-1}(t-\frac{t^{2}}{T})\right) \varvec{\varSigma }_{0} = \left( \frac{T(T-1)}{2}-\frac{(2T-1)(T-1)}{6}\right) \varvec{\varSigma }_{0}\\ =&\frac{(T+1)(T-1)}{6}\varvec{\varSigma }_{0}. \end{aligned}$$

In Juodis (2016) it is shown that the hessian matrix is of the following form (if we neglect terms that evaluated at the true values $\varvec{\kappa }_{0}$ have expectation $\mathbf {O}$):

25 $$\begin{aligned}&\varvec{\mathcal {H}}^{N}(\varvec{\kappa })\nonumber \\&\quad =-N\left( \begin{array}{ccc} \varvec{R}_{N}\otimes \varvec{\varSigma }^{-1}+\varvec{P}_{N}\otimes \varvec{\varTheta }^{-1} &{} (\varvec{Q}_{N}\varvec{\varSigma }^{-1} \otimes \varvec{\varSigma }^{-1}){{\mathrm{\mathbf {D}}}}_{m} &{} (\varvec{N}_{N}\varvec{\varTheta }^{-1} \otimes \varvec{\varTheta }^{-1}){{\mathrm{\mathbf {D}}}}_{m} \\ {{\mathrm{\mathbf {D}}}}_{m}'(\varvec{\varSigma }^{-1}\varvec{Q}_{N}' \otimes \varvec{\varSigma }^{-1}) &{} \frac{(T-1)}{2}{{\mathrm{\mathbf {D}}}}_{m}'(\varvec{\varSigma }^{-1}\otimes \varvec{\varSigma }^{-1}){{\mathrm{\mathbf {D}}}}_{m} &{} \mathbf {O}_{p} \\ {{\mathrm{\mathbf {D}}}}_{m}'(\varvec{\varTheta }^{-1}\varvec{N}_{N}' \otimes \varvec{\varTheta }^{-1}) &{} \mathbf {O}_{p} &{} \frac{1}{2}{{\mathrm{\mathbf {D}}}}_{m}'(\varvec{\varTheta }^{-1}\otimes \varvec{\varTheta }^{-1}){{\mathrm{\mathbf {D}}}}_{m} \\ \end{array} \right) .\nonumber \\ \end{aligned}$$

Plugging in these expressions into the formula for the hessian and evaluating it at the true value $\varvec{\kappa }_{0}$:

26 $$\begin{aligned} {\varvec{\mathcal {H}}}_{\ell }=\frac{(T-1)}{2}\left( \begin{array}{c@{\quad }c@{\quad }c} T(\varvec{\varSigma }_{0} \otimes \varvec{\varSigma }_{0}^{-1}) &{}-(\varvec{I}_{m} \otimes \varvec{\varSigma }_{0}^{-1}){{\mathrm{\mathbf {D}}}}_{m} &{} (\varvec{I}_{m} \otimes \varvec{\varSigma }_{0}^{-1}){{\mathrm{\mathbf {D}}}}_{m}\\ -{{\mathrm{\mathbf {D}}}}_{m}'(\varvec{I}_{m} \otimes \varvec{\varSigma }_{0}^{-1}) &{} {{\mathrm{\mathbf {D}}}}_{m}'(\varvec{\varSigma }_{0}^{-1} \otimes \varvec{\varSigma }_{0}^{-1}){{\mathrm{\mathbf {D}}}}_{m} &{} \mathbf {O}_{p} \\ {{\mathrm{\mathbf {D}}}}_{m}'(\varvec{I}_{m} \otimes \varvec{\varSigma }_{0}^{-1}) &{} \mathbf {O}_{p} &{} \frac{1}{T-1}{{\mathrm{\mathbf {D}}}}_{m}'(\varvec{\varSigma }_{0}^{-1} \otimes \varvec{\varSigma }_{0}^{-1}){{\mathrm{\mathbf {D}}}}_{m}\\ \end{array} \right) .\nonumber \\ \end{aligned}$$

Using the formula for the determinant of partitioned matrix, that:

27 $$\begin{aligned} |{\varvec{\mathcal {H}}}_{\ell }|\propto \left( |\varvec{H}|\times \left| \frac{1}{T-1}{{\mathrm{\mathbf {D}}}}_{m}'(\varvec{\varSigma }_{0}^{-1} \otimes \varvec{\varSigma }_{0}^{-1}){{\mathrm{\mathbf {D}}}}_{m}\right| \times |{{\mathrm{\mathbf {D}}}}_{m}'(\varvec{\varSigma }_{0}^{-1} \otimes \varvec{\varSigma }_{0}^{-1}){{\mathrm{\mathbf {D}}}}_{m}|\right) , \end{aligned}$$

where $\propto$ is the proportionality sign. For future reference, define

$$\begin{aligned} \varvec{H}= & {} ({\text {E}}[\varvec{R}_{N}]\otimes \varvec{\varSigma }_{0}^{-1}+{\text {E}}[\varvec{P}_{N}]\otimes \varvec{\varTheta }_{0}^{-1})\\&-\left[ \frac{1}{T-1}\left( \left( {\text {E}}[\varvec{Q}_{N}(\varvec{\kappa }_{0})]\varvec{\varSigma }_{0}^{-1}\right) \otimes \left( {\text {E}}[\varvec{Q}_{N}(\varvec{\kappa }_{0})]\varvec{\varSigma }_{0}^{-1}\right) '\right) \right. \\&\quad \left. +\left( \left( {\text {E}}[\varvec{N}_{N}(\varvec{\kappa }_{0})]\varvec{\varTheta }_{0}^{-1}\right) \otimes \left( {\text {E}}[\varvec{N}_{N}(\varvec{\kappa }_{0})]\varvec{\varTheta }_{0}^{-1}\right) '\right) \right] {{\mathrm{\mathbf {K}}}}_{m}\\&-\left[ \frac{1}{T-1}\left( \left( {\text {E}}[\varvec{Q}_{N}(\varvec{\kappa }_{0})]\varvec{\varSigma }_{0}^{-1}{\text {E}}[\varvec{Q}_{N}(\varvec{\kappa }_{0})]'\right) \otimes \varvec{\varSigma }_{0}^{-1}\right) \right. \\&\quad \left. +\left( \left( {\text {E}}[\varvec{N}_{N}(\varvec{\kappa }_{0})]\varvec{\varTheta }_{0}^{-1}{\text {E}}[\varvec{N}_{N}(\varvec{\kappa }_{0})]'\right) \otimes \varvec{\varTheta }_{0}^{-1}\right) \right] . \end{aligned}$$

Thus, $\varvec{H}$ in this case given by:

$$\begin{aligned} \varvec{H}&=T\left( (\varvec{\varSigma }_{0}\otimes \varvec{\varSigma }_{0}^{-1})-(\varvec{I}_{m} \otimes \varvec{\varSigma }_{0}^{-1}){{\mathrm{\mathbf {D}}}}_{m}{{\mathrm{\mathbf {D}}}}_{m}^{+}(\varvec{\varSigma }_{0} \otimes \varvec{\varSigma }_{0})({{\mathrm{\mathbf {D}}}}_{m}{{\mathrm{\mathbf {D}}}}_{m}^{+})'(\varvec{I}_{m} \otimes \varvec{\varSigma }_{0}^{-1})\right) \\&=T\left( (\varvec{\varSigma }_{0}\otimes \varvec{\varSigma }_{0}^{-1})-(\varvec{I}_{m} \otimes \varvec{\varSigma }_{0}^{-1}){{\mathrm{\mathbf {D}}}}_{m}{{\mathrm{\mathbf {D}}}}_{m}^{+}(\varvec{\varSigma }_{0} \otimes \varvec{\varSigma }_{0})(\varvec{I}_{m} \otimes \varvec{\varSigma }_{0}^{-1})\right) \\&=T\left( (\varvec{\varSigma }_{0}\otimes \varvec{\varSigma }_{0}^{-1})-\frac{1}{2}(\varvec{I}_{m} \otimes \varvec{\varSigma }_{0}^{-1})(\varvec{I}_{m}+{{\mathrm{\mathbf {K}}}}_{m})(\varvec{\varSigma }_{0} \otimes \varvec{\varSigma }_{0})(\varvec{I}_{m} \otimes \varvec{\varSigma }_{0}^{-1})\right) \\&=\frac{T}{2}\left( (\varvec{\varSigma }_{0}\otimes \varvec{\varSigma }_{0}^{-1})-(\varvec{I}_{m} \otimes \varvec{\varSigma }_{0}^{-1}){{\mathrm{\mathbf {K}}}}_{m}(\varvec{\varSigma }_{0} \otimes \varvec{\varSigma }_{0})(\varvec{I}_{m} \otimes \varvec{\varSigma }_{0}^{-1})\right) \\&=\frac{T}{2}\left( (\varvec{\varSigma }_{0}\otimes \varvec{\varSigma }_{0}^{-1})-{{\mathrm{\mathbf {K}}}}_{m}\right) . \end{aligned}$$

Then by means of the matrix determinant Lemma:

$$\begin{aligned} |\varvec{H}|&\propto |\varvec{I}_{m^{2}}-(\varvec{I}_{m}\otimes \varvec{\varSigma }_{0}^{-1}){{\mathrm{\mathbf {K}}}}_{m}(\varvec{\varSigma }_{0}\otimes \varvec{I}_{m})||{{\mathrm{\mathbf {K}}}}_{m}|\\&\propto |\varvec{I}_{m^{2}}-(\varvec{I}_{m}\otimes \varvec{\varSigma }_{0}^{-1}){{\mathrm{\mathbf {K}}}}_{m}(\varvec{\varSigma }_{0}\otimes \varvec{I}_{m})|\\&\propto |\varvec{I}_{m^{2}}-{{\mathrm{\mathbf {K}}}}_{m}|=0, \end{aligned}$$

where in the first line we used the fact that ${{\mathrm{\mathbf {K}}}}_{m}={{\mathrm{\mathbf {K}}}}_{m}^{-1}$ and the second line follows from the fact that $|{{\mathrm{\mathbf {K}}}}_{m}|=(-1)^{0.5 m(m-1)}$, while $|\varvec{I}_{m^{2}}-{{\mathrm{\mathbf {K}}}}_{m}|=0$ follows trivially from the fact that ${\text {rk}}\,{(\varvec{I}_{m^{2}}-{{\mathrm{\mathbf {K}}}}_{m})}=0.5m(m-1)$. Hence we have proved that the ${\varvec{\mathcal {H}}}_{\ell }$ matrix is not invertible.

#### Cointegration and $T=2$

##### Proof of Proposition 1

Using derivations of previous section it follows that:

$$\begin{aligned}&\displaystyle {\text {E}}[\varvec{P}_{N}]={\text {E}}[\varvec{R}_{N}]=\frac{1}{2}\varvec{\varPsi }_{0}\\&\displaystyle {\text {E}}[\varvec{N}_{N}(\varvec{\kappa }_{0})]=\frac{1}{2}\varvec{\varTheta }_{0}\\&\displaystyle {\text {E}}[\varvec{Q}_{N}(\varvec{\kappa }_{0})]=-\frac{1}{2}\varvec{\varSigma }_{0} \end{aligned}$$

Corresponding $$\varvec{H}$$ matrix:

$$\begin{aligned} \varvec{H}=&\,\frac{1}{2}\varvec{\varPsi }_{0}\otimes \left( \varvec{\varTheta }_{0}^{-1}+\varvec{\varSigma }_{0}^{-1}\right) \\&- \frac{1}{2}\left( \varvec{I}_{m} \otimes \varvec{\varSigma }_{0}^{-1}\right) {{\mathrm{\mathbf {D}}}}_{m}{{\mathrm{\mathbf {D}}}}_{m}^{+}\left( \varvec{\varSigma }_{0} \otimes \varvec{\varSigma }_{0}\right) \left( {{\mathrm{\mathbf {D}}}}_{m}{{\mathrm{\mathbf {D}}}}_{m}^{+}\right) '\left( \varvec{I}_{m} \otimes \varvec{\varSigma }_{0}^{-1}\right) \\&-\frac{1}{2}\left( \varvec{I}_{m} \otimes \varvec{\varTheta }_{0}^{-1}\right) {{\mathrm{\mathbf {D}}}}_{m}{{\mathrm{\mathbf {D}}}}_{m}^{+}(\varvec{\varTheta }_{0} \otimes \varvec{\varTheta }_{0})\left( {{\mathrm{\mathbf {D}}}}_{m}{{\mathrm{\mathbf {D}}}}_{m}^{+}\right) '\left( \varvec{I}_{m} \otimes \varvec{\varTheta }_{0}^{-1}\right) \\ =&\frac{1}{2}\varvec{\varPsi }_{0}\otimes \left( \varvec{\varTheta }_{0}^{-1}+\varvec{\varSigma }_{0}^{-1}\right) -\frac{1}{2}(\varvec{\varSigma }_{0} \otimes \varvec{I}_{m})\left( {{\mathrm{\mathbf {D}}}}_{m}{{\mathrm{\mathbf {D}}}}_{m}^{+}\right) '\left( \varvec{I}_{m} \otimes \varvec{\varSigma }_{0}^{-1}\right) \\&-\frac{1}{2}(\varvec{\varTheta }_{0} \otimes \varvec{I}_{m})({{\mathrm{\mathbf {D}}}}_{m}{{\mathrm{\mathbf {D}}}}_{m}^{+})'(\varvec{I}_{m} \otimes \varvec{\varTheta }_{0}^{-1})\\&\propto \varvec{\varPsi }_{0}\otimes \left( \varvec{\varTheta }_{0}^{-1}+\varvec{\varSigma }_{0}^{-1}\right) -{{\mathrm{\mathbf {K}}}}_{m}-\frac{1}{2}\left( \varvec{\varTheta }_{0} \otimes \varvec{\varTheta }_{0}^{-1}\right) -\frac{1}{2}\left( \varvec{\varSigma }_{0} \otimes \varvec{\varSigma }_{0}^{-1}\right) \\&\propto \left( \varvec{\varSigma }_{0}\otimes \varvec{\varTheta }_{0}^{-1}-{{\mathrm{\mathbf {K}}}}_{m}\right) + \left( \varvec{\varTheta }_{0}\otimes \varvec{\varSigma }_{0}^{-1}-{{\mathrm{\mathbf {K}}}}_{m}\right) . \end{aligned}$$

We can express $\varvec{\varTheta }_{0}=\varvec{\varSigma }_{0}+\varvec{a}\varvec{a}'$ with $\varvec{a}$ being of rank *r*. Then:

$$\begin{aligned} \varvec{H}&\propto \left( \varvec{I}_{m}\otimes \varvec{\varTheta }_{0}^{-1}\right) \left( \varvec{\varSigma }_{0}\otimes \varvec{\varSigma }_{0}+\varvec{\varTheta }_{0}\otimes \varvec{\varTheta }_{0}-2 (\varvec{\varSigma }_{0}\otimes \varvec{\varTheta }_{0}){{\mathrm{\mathbf {K}}}}_{m}\right) \left( \varvec{I}_{m}\otimes \varvec{\varSigma }_{0}^{-1}\right) \\&=\left( \varvec{I}_{m}\otimes \varvec{\varTheta }_{0}^{-1}\right) \left( 2(\varvec{\varSigma }_{0}\otimes \varvec{\varSigma }_{0})(\varvec{I}_{m^{2}}-{{\mathrm{\mathbf {K}}}}_{m})\right. \\&\left. +\,(\varvec{\varSigma }_{0}\otimes \varvec{a}\varvec{a}')(\varvec{I}_{m^{2}}-{{\mathrm{\mathbf {K}}}}_{m})+\varvec{a}\varvec{a}'\otimes \varvec{a}\varvec{a}'\right) (\varvec{I}_{m}\otimes \varvec{\varSigma }_{0}^{-1})\\&=\left( \varvec{I}_{m}\otimes \varvec{\varTheta }_{0}^{-1}\right) \left( (2\varvec{\varSigma }_{0}\otimes \varvec{\varSigma }_{0}{+}\varvec{\varSigma }_{0}\otimes \varvec{a}\varvec{a}')(\varvec{I}_{m^{2}}{-}{{\mathrm{\mathbf {K}}}}_{m}){+}\varvec{a}\varvec{a}'\otimes \varvec{a}\varvec{a}'\right) (\varvec{I}_{m}\otimes \varvec{\varSigma }_{0}^{-1}). \end{aligned}$$

Thus using basic inequalities for the rank of matrix sum we can deduce that ${\text {rk}}\,\varvec{H}$ is no greater than $0.5m(m-1)+r^{2}$, due to the full rank of $(2\varvec{\varSigma }_{0}\otimes \varvec{\varSigma }_{0}+\varvec{\varSigma }_{0}\otimes \varvec{a}\varvec{a}')$ matrix.

### Monte Carlo results

#### Robustness check: spatial dependence

To allow for possible spatial dependence, the $\varvec{\varepsilon }_{i,t}$ for all *t* are generated with Spatial MA process:

$$\begin{aligned} \varvec{\varepsilon }_{i,t}&=\theta \sum _{j=1}^{N}\omega _{i,j}\varvec{\zeta }_{j,t}+\varvec{\zeta }_{i,t},t>0\\ \varvec{\zeta }_{i,t}&\sim N(\varvec{0}_{2},\varvec{\varSigma }),\\ \varvec{\zeta }_{i,0}&\sim N\left( \varvec{0}_{2},\sum _{j=0}^{M}\varvec{\varPhi }^{j}\varvec{\varSigma }\varvec{\varPhi }^{j'}\right) . \end{aligned}$$

The $\theta$ parameter controls the degree of cross-sectional dependence between units. For $\theta =0$ we have i.i.d. dataset, while for $\theta \ne 0$ the cross-sectional units are *weakly* correlated. To illustrate the effect of spatial dependence we consider one value for $\theta$, namely $\theta =0.5$. Spatial correlation matrix $\varvec{W}_{N}$ with a typical (*i*, *j*) element given by $\omega _{i,j}$ is assumed to be **1 ahead - 1 behind** circular, so that every individual *i* is directly linked only with individuals $i-1$ and $i+1$.^35^ The particular choice of the spatial matrix $\varvec{W}_{N}$ is motivated by the study in Baltagi et al. (2007), where in the context of the panel unit root testing it is shown that the tests are mostly distorted for this choice of spatial matrix.^36^ The results are presented at the bottom of Tables 6, 7, and 8.

Evidence of the uniform upward shift in the size can be observed when designs with spatial dependence ($\theta = 0.5$) are considered. This upward movement does not come as a surprise because similar patterns have been documented in the panel unit root testing literature. However, the same conclusion can not be reached regarding the test power, as for most scenarios it changes marginally and does not show any clear patterns in terms of magnitude and direction. More importantly, major size distortions *do* not disappear for $N=500$, thus as can be expected the fact that we use the variance-covariance matrix that ignores the presence of spatial dependence has a pronounced result. On the other hand, the fact that by ignoring the spatial dependence the rank based test statistic is only mildly oversized, suggests that the procedure developed in this paper is relatively robust to deviations from the i.i.d. assumption.

#### Tables

See Tables 5, 6, 7, 8, 9, 10, and 11.

**Table 5:** MC results for LR test based on TML

Test	$\{T,\tau \}$ $\backslash \lambda$	$-$.70	$-$.30	$-$.10	$-$.05	$-$.01	$-$.005	.00
rk-J	$\{ 3 ; 1 \}$					0.90	0.49	0.04
	$\{ 3 ; 5 \}$	0.27	0.35	0.94	0.97	0.70	0.27	0.03
	$\{ 5 ; 1 \}$						0.77	0.03
	$\{ 5 ; 5 \}$		0.91	0.98		0.93	0.59	0.05
	$\{ 7 ; 1 \}$						0.93	0.05
	$\{ 7 ; 5 \}$			0.99	0.99	0.98	0.78	0.04
TML	$\{ 3 ; 1 \}$	0.96	0.65	0.04	0.01	0.01	0.01	0.01
	$\{ 3 ; 5 \}$		0.88	0.06	0.02	0.01	0.01	0.01
	$\{ 5 ; 1 \}$		0.95	0.13	0.03	0.03	0.02	0.02
	$\{ 5 ; 5 \}$			0.14	0.05	0.03	0.03	0.03
	$\{ 7 ; 1 \}$			0.46	0.08	0.01	0.01	0.01
	$\{ 7 ; 5 \}$			0.51	0.09	0.01	0.02	0.02

Results are based on 500 replications. Here $\alpha =-0.1$, while $\varvec{\varUpsilon }^{(1)}$ is used for initialization and $N=750$. Empty entries correspond to $100\%$ empirical rejection frequencies. As starting values for TML estimators we use the bias corrected Fixed Effects estimators as define in Table 2

**Table 6:** Eigenvalues

$\alpha$	$\varvec{\varUpsilon }^{(q)}$	$\{T,\tau \}$ $\backslash \lambda$	$-$0.70	$-$0.30	$-$0.10	$-$0.05	$-$0.01	$-$0.005	0.00
$-$0.1	1	$\{ 3 ; 1 \}$	0.07	0.06	0.04	0.03	0.02	0.02	0.00
		$\{ 3 ; 5 \}$	0.03	0.03	0.03	0.02	0.02	0.01	0.00
		$\{ 5 ; 1 \}$	0.08	0.06	0.04	0.03	0.02	0.02	0.00
		$\{ 5 ; 5 \}$	0.07	0.04	0.03	0.03	0.02	0.01	0.00
		$\{ 7 ; 1 \}$	0.08	0.06	0.04	0.03	0.02	0.02	0.00
		$\{ 7 ; 5 \}$	0.09	0.05	0.03	0.03	0.02	0.01	0.00
	2	$\{ 3 ; 1 \}$	0.08	0.06	0.04	0.04	0.02	0.02	0.00
		$\{ 3 ; 5 \}$	0.08	0.06	0.04	0.04	0.02	0.02	0.00
		$\{ 5 ; 1 \}$	0.08	0.06	0.04	0.04	0.03	0.02	0.00
		$\{ 5 ; 5 \}$	0.08	0.06	0.04	0.04	0.02	0.02	0.00
		$\{ 7 ; 1 \}$	0.08	0.06	0.04	0.04	0.03	0.02	0.00
		$\{ 7 ; 5 \}$	0.08	0.06	0.04	0.04	0.02	0.02	0.00
	3	$\{ 3 ; 1 \}$	0.10	0.08	0.05	0.04	0.03	0.02	0.00
		$\{ 3 ; 5 \}$	0.17	0.13	0.08	0.06	0.02	0.01	0.00
		$\{ 5 ; 1 \}$	0.09	0.07	0.05	0.04	0.03	0.02	0.00
		$\{ 5 ; 5 \}$	0.14	0.12	0.08	0.06	0.02	0.01	0.00
		$\{ 7 ; 1 \}$	0.09	0.07	0.05	0.04	0.03	0.02	0.00
		$\{ 7 ; 5 \}$	0.12	0.11	0.08	0.06	0.02	0.01	0.00
	1	$\{ 3 ; 1 \}$	0.13	0.05	0.03	0.03	0.02	0.02	0.00
		$\{ 3 ; 5 \}$	0.18	0.06	0.00	0.00	0.01	0.00	0.00
		$\{ 5 ; 1 \}$	0.14	0.06	0.03	0.03	0.02	0.02	0.00
		$\{ 5 ; 5 \}$	0.21	0.07	0.02	0.01	0.00	0.00	0.00
		$\{ 7 ; 1 \}$	0.14	0.06	0.03	0.03	0.02	0.01	0.00
		$\{ 7 ; 5 \}$	0.19	0.08	0.03	0.02	0.01	0.01	0.00
$-$0.5	2	$\{ 3 ; 1 \}$	0.12	0.06	0.04	0.02	0.02	0.01	0.00
		$\{ 3 ; 5 \}$	0.12	0.06	0.04	0.02	0.02	0.01	0.00
		$\{ 5 ; 1 \}$	0.12	0.06	0.04	0.02	0.02	0.01	0.00
		$\{ 5 ; 5 \}$	0.12	0.06	0.04	0.02	0.02	0.01	0.00
		$\{ 7 ; 1 \}$	0.12	0.06	0.04	0.02	0.02	0.01	0.00
		$\{ 7 ; 5 \}$	0.12	0.06	0.04	0.02	0.02	0.01	0.00
	3	$\{ 3 ; 1 \}$	0.15	0.08	0.03	0.02	0.02	0.01	0.00
		$\{ 3 ; 5 \}$	0.27	0.12	0.06	0.04	0.02	0.01	0.00
		$\{ 5 ; 1 \}$	0.13	0.07	0.03	0.02	0.02	0.01	0.00
		$\{ 5 ; 5 \}$	0.17	0.09	0.05	0.04	0.02	0.01	0.00
		$\{ 7 ; 1 \}$	0.13	0.06	0.03	0.02	0.02	0.01	0.00
		$\{ 7 ; 5 \}$	0.14	0.07	0.04	0.03	0.02	0.01	0.00

The minima of modulus’ of eigenvalues of $\overline{\Delta {}\varvec{y}_{i,t}\varvec{y}_{i,t-1}'}_{T}$ for $N=200{,}000$

**Table 7:** Eigenvalues

$\alpha$	$\varvec{\varUpsilon }^{(q)}$	$\{T,\tau \}$ $\backslash \lambda$	$-$0.70	$-$0.30	$-$0.10	$-$0.05	$-$0.01	$-$0.005	0.00
$-$0.1	1	$\{ 3 ; 1 \}$	0.18	0.31	0.51	0.51	0.46	0.46	0.45
		$\{ 3 ; 5 \}$	0.04	0.09	0.43	0.40	0.29	0.28	0.27
		$\{ 5 ; 1 \}$	0.42	0.66	1.03	1.01	0.93	0.92	0.91
		$\{ 5 ; 5 \}$	0.41	0.43	0.99	0.81	0.59	0.57	0.54
		$\{ 7 ; 1 \}$	0.68	1.05	1.57	1.52	1.39	1.38	1.37
		$\{ 7 ; 5 \}$	0.92	1.09	1.58	1.25	0.91	0.87	0.84
	2	$\{ 3 ; 1 \}$	0.22	0.36	0.54	0.53	0.50	0.50	0.50
		$\{ 3 ; 5 \}$	0.22	0.36	0.54	0.53	0.50	0.50	0.50
		$\{ 5 ; 1 \}$	0.43	0.72	1.08	1.07	1.01	1.00	1.00
		$\{ 5 ; 5 \}$	0.44	0.72	1.08	1.07	1.01	1.01	1.01
		$\{ 7 ; 1 \}$	0.65	1.09	1.61	1.60	1.51	1.51	1.51
		$\{ 7 ; 5 \}$	0.65	1.09	1.62	1.60	1.51	1.51	1.51
	3	$\{ 3 ; 1 \}$	0.28	0.46	0.61	0.61	0.61	0.61	0.62
		$\{ 3 ; 5 \}$	0.46	0.74	0.88	0.90	1.01	1.04	1.07
		$\{ 5 ; 1 \}$	0.49	0.85	1.20	1.21	1.20	1.21	1.22
		$\{ 5 ; 5 \}$	0.70	1.29	1.72	1.78	1.95	2,00	2,05
		$\{ 7 ; 1 \}$	0.68	1.22	1.79	1.81	1.78	1.79	1.80
		$\{ 7 ; 5 \}$	0.85	1.73	2,52	2,64	2,83	2,89	2,95
	1	$\{ 3 ; 1 \}$	0.36	0.15	0.09	0.09	0.09	0.09	0.09
		$\{ 3 ; 5 \}$	0.50	0.25	0.03	0.02	0.04	0.04	0.05
		$\{ 5 ; 1 \}$	0.78	0.31	0.20	0.19	0.19	0.19	0.19
		$\{ 5 ; 5 \}$	1.18	0.64	0.15	0.05	0.01	0.01	0.01
		$\{ 7 ; 1 \}$	1.02	0.48	0.31	0.30	0.30	0.31	0.31
		$\{ 7 ; 5 \}$	1.61	0.88	0.27	0.15	0.10	0.10	0.10
$-$0.5	2	$\{ 3 ; 1 \}$	0.30	0.16	0.12	0.12	0.12	0.12	0.12
		$\{ 3 ; 5 \}$	0.30	0.16	0.12	0.12	0.12	0.12	0.12
		$\{ 5 ; 1 \}$	0.60	0.31	0.24	0.24	0.24	0.24	0.24
		$\{ 5 ; 5 \}$	0.60	0.31	0.24	0.24	0.24	0.24	0.24
		$\{ 7 ; 1 \}$	0.90	0.47	0.36	0.35	0.36	0.36	0.36
		$\{ 7 ; 5 \}$	0.90	0.47	0.36	0.36	0.36	0.36	0.36
	3	$\{ 3 ; 1 \}$	0.36	0.22	0.18	0.18	0.18	0.18	0.18
		$\{ 3 ; 5 \}$	0.75	0.60	0.44	0.42	0.41	0.41	0.41
		$\{ 5 ; 1 \}$	0.65	0.41	0.33	0.32	0.32	0.32	0.32
		$\{ 5 ; 5 \}$	0.96	1.04	0.74	0.68	0.63	0.63	0.62
		$\{ 7 ; 1 \}$	0.93	0.55	0.46	0.45	0.44	0.44	0.44
		$\{ 7 ; 5 \}$	1.18	1.20	0.95	0.85	0.79	0.78	0.78

The minima of modulus’ of eigenvalues of $\overline{\varvec{u}_{i,t-1}\varvec{y}_{i,t-1}'}_{T}$ for $N=200{,}000$

**Table 8:** Monte Carlo results for $\alpha =-0.1$. $\varvec{\varUpsilon }^{(1){-}(3)}$

$\theta$	$\{N,T,\tau \}$ $\backslash \lambda$	$\varvec{\varUpsilon }^{(1)}$							$\varvec{\varUpsilon }^{(2)}$							$\varvec{\varUpsilon }^{(3)}$						
		$-$.70	$-$.30	$-$.10	$-$.05	$-$.01	$-$.005	.00	$-$.70	$-$.30	$-$.10	$-$.05	$-$.01	$-$.005	.00	$-$.70	$-$.30	$-$.10	$-$.05	$-$.01	$-$.005	.00
0.0	50, 3, 1	.83	.81	.63	.36	.07	.04	.03	.90	.86	.64	.37	.07	.04	.03	.94	.90	.71	.42	.08	.05	.03
	50, 3, 5	.20	.20	.21	.14	.04	.03	.02	.62	.57	.37	.21	.05	.04	.03	.94	.88	.69	.43	.09	.05	.03
	50, 5, 1	.98	.95	.91	.75	.16	.08	.04		.99	.93	.76	.17	.08	.04		.99	.96	.81	.19	.09	.04
	50, 5, 5	.68	.40	.40	.33	.08	.04	.03	.88	.83	.69	.51	.12	.07	.03		.99	.93	.79	.20	.10	.04
	50, 7, 1		.99	.97	.92	.29	.12	.05			.99	.93	.30	.13	.05				.95	.31	.14	.05
	50, 7, 5	.97	.63	.52	.48	.15	.07	.04	.95	.92	.84	.71	.22	.11	.05			.98	.93	.31	.14	.05
	250, 3, 1	.99				.41	.16	.04				.99	.41	.16	.04					.44	.17	.04
	250, 3, 5	.21	.26	.65	.66	.17	.06	.02			.98	.90	.30	.11	.03				.99	.44	.17	.04
	250, 5, 1					.77	.34	.04					.77	.34	.05					.78	.35	.05
	250, 5, 5	.97	.65	.80	.85	.46	.17	.03					.65	.28	.04					.76	.34	.05
	250, 7, 1					.92	.51	.05					.92	.51	.05					.93	.52	.05
	250, 7, 5		.94	.88	.90	.66	.29	.04					.84	.43	.05					.91	.49	.05
	500, 3, 1					.75	.32	.04					.75	.33	.04					.77	.34	.05
	500, 3, 5	.24	.31	.85	.91	.45	.16	.03					.62	.26	.04					.75	.33	.05
	500, 5, 1					.97	.62	.05					.97	.61	.05					.97	.62	.05
	500, 5, 5		.80	.93	.96	.79	.39	.03					.93	.53	.05					.97	.60	.05
	500, 7, 1						.80	.05						.80	.05						.81	.05
	500, 7, 5		.99	.97	.98	.90	.58	.05					.99	.72	.05						.78	.05
0.5	50, 3, 1	.84	.82	.63	.37	.09	.07	.05	.89	.85	.64	.37	.09	.06	.05	.93	.88	.69	.41	.10	.07	.05
	50, 3, 5	.27	.27	.27	.18	.06	.05	.04	.67	.61	.40	.24	.07	.05	.04	.93	.86	.67	.41	.10	.07	.05
	50, 5, 1	.99	.97	.91	.74	.20	.11	.07	.99	.99	.93	.75	.20	.11	.07		.99	.95	.79	.21	.12	.07
	50, 5, 5	.75	.50	.49	.39	.11	.07	.05	.91	.86	.73	.53	.15	.09	.06		.99	.93	.77	.21	.12	.07
	50, 7, 1		.99	.98	.91	.31	.16	.08			.99	.92	.32	.16	.08			.99	.94	.33	.17	.08
	50, 7, 5	.98	.72	.63	.56	.19	.11	.07	.97	.94	.87	.75	.25	.14	.07			.98	.92	.32	.17	.08
	250, 3, 1				.99	.42	.19	.06				.99	.42	.19	.07				.99	.44	.20	.07
	250, 3, 5	.40	.47	.80	.78	.24	.11	.04			.98	.92	.33	.15	.05				.99	.43	.20	.07
	250, 5, 1					.75	.36	.08					.74	.36	.08					.75	.37	.08
	250, 5, 5	.98	.81	.91	.93	.55	.23	.05					.65	.31	.07					.74	.36	.08
	250, 7, 1					.90	.52	.09					.90	.51	.09					.91	.52	.08
	250, 7, 5		.97	.95	.96	.74	.38	.07					.84	.45	.08					.89	.50	.08
	500, 3, 1					.72	.34	.08					.72	.34	.08					.73	.35	.08
	500, 3, 5	.50	.61	.95	.97	.54	.22	.06					.63	.28	.07					.72	.34	.07
	500, 5, 1					.95	.61	.08					.95	.60	.08					.96	.61	.08
	500, 5, 5		.92	.98	.99	.86	.47	.07					.92	.54	.08					.95	.60	.08
	500, 7, 1						.77	.08						.77	.08						.78	.08
	500, 7, 5			.99	.99	.96	.65	.07					.99	.71	.08					.99	.76	.08

$\theta =0$ corresponds to the model without spatial dependence (Sect. 4, while $\theta =0.5$ the model with spatial dependence of circular form from 6. Empty entries correspond to $100\%$ empirical rejection frequencies

**Table 9:** Monte Carlo results for $\alpha =-0.1$. $\varvec{\varUpsilon }^{(4),(6)}$

$\theta$	$\{N,T,\tau \}$ $\backslash \lambda$	$\varvec{\varUpsilon }^{(4)}$							$\varvec{\varUpsilon }^{(5)}$						
		$-$.70	$-$.30	$-$.10	$-$.05	$-$.01	$-$.005	.00	$-$.70	$-$.30	$-$.10	$-$.05	$-$.01	$-$.005	.00
0.0	50, 3, 1	.92	.89	.66	.38	.07	.04	.03	.88	.84	.64	.37	.07	.04	.03
	50, 3, 5	.79	.81	.52	.21	.04	.03	.03	.36	.39	.30	.19	.05	.03	.02
	50, 5, 1		.99	.94	.76	.17	.08	.04	.99	.97	.92	.76	.17	.08	.04
	50, 5, 5	.97	.98	.85	.50	.09	.05	.03	.66	.59	.56	.45	.11	.06	.03
	50, 7, 1			.99	.93	.29	.12	.05		.99	.98	.92	.30	.13	.05
	50, 7, 5	.99		.95	.71	.17	.09	.04	.84	.73	.70	.63	.21	.10	.04
	250, 3, 1					.43	.16	.04				.99	.41	.16	.04
	250, 3, 5				.94	.22	.08	.03	.74	.85	.92	.85	.29	.11	.03
	250, 5, 1					.79	.35	.05					.77	.34	.05
	250, 5, 5				.99	.52	.19	.04	.95	.93	.97	.97	.63	.27	.04
	250, 7, 1					.93	.52	.05					.92	.51	.05
	250, 7, 5					.71	.32	.04	.99	.97	.99	.99	.83	.42	.05
	500, 3, 1					.77	.33	.04					.75	.32	.04
	500, 3, 5					.50	.18	.03	.89	.96	.99	.99	.62	.25	.04
	500, 5, 1					.98	.63	.05					.97	.61	.05
	500, 5, 5					.81	.40	.04	.99	.99			.92	.52	.04
	500, 7, 1						.81	.05						.80	.05
	500, 7, 5					.92	.60	.05					.99	.72	.05
0.5	50, 3, 1	.87	.84	.63	.37	.10	.06	.05	.87	.84	.63	.37	.10	.06	.05
	50, 3, 5	.44	.45	.35	.22	.07	.05	.04	.44	.45	.35	.22	.07	.05	.04
	50, 5, 1	.99	.98	.92	.74	.20	.11	.07	.99	.98	.92	.74	.20	.11	.07
	50, 5, 5	.75	.68	.63	.49	.15	.09	.06	.75	.68	.63	.49	.15	.09	.06
	50, 7, 1			.98	.92	.32	.16	.08			.98	.92	.32	.16	.08
	50, 7, 5	.89	.81	.77	.68	.24	.13	.07	.89	.81	.77	.68	.24	.13	.07
	250, 3, 1				.99	.42	.19	.07				.99	.42	.19	.07
	250, 3, 5	.87	.93	.95	.88	.32	.15	.05	.87	.93	.95	.88	.32	.15	.05
	250, 5, 1					.74	.36	.08					.74	.36	.08
	250, 5, 5	.98	.97	.99	.99	.64	.30	.07	.98	.97	.99	.99	.64	.30	.07
	250, 7, 1					.90	.51	.09					.90	.51	.09
	250, 7, 5		.99	.99		.83	.45	.08		.99	.99		.83	.45	.08
	500, 3, 1					.72	.34	.08					.72	.34	.08
	500, 3, 5	.97	.99		.99	.62	.28	.06	.97	.99		.99	.62	.28	.06
	500, 5, 1					.95	.60	.08					.95	.60	.08
	500, 5, 5					.91	.54	.08					.91	.54	.08
	500, 7, 1						.77	.08						.77	.08
	500, 7, 5					.98	.71	.08					.98	.71	.08

See Table 8

**Table 10:** Monte Carlo results for $\alpha =-0.5$. $\varvec{\varUpsilon }^{(1){-}(3)}$

$\theta$	$\{N,T,\tau \}$ $\backslash \lambda$	$\varvec{\varUpsilon }^{(1)}$							$\varvec{\varUpsilon }^{(2)}$							$\varvec{\varUpsilon }^{(3)}$						
		$-$.70	$-$.30	$-$.10	$-$.05	$-$.01	$-$.005	.00	$-$.70	$-$.30	$-$.10	$-$.05	$-$.01	$-$.005	.00	$-$.70	$-$.30	$-$.10	$-$.05	$-$.01	$-$.005	.00
0.0	50, 3, 1	.99	.88	.59	.36	.08	.05	.04	.99	.97	.72	.45	.10	.06	.04			.84	.54	.12	.07	.03
	50, 3, 5	.94	.34	.09	.08	.03	.02	.02	.86	.64	.36	.23	.08	.05	.03	.99	.97	.83	.54	.13	.08	.04
	50, 5, 1		.99	.91	.79	.25	.13	.06			.97	.85	.29	.15	.07			.99	.89	.30	.16	.06
	50, 5, 5		.88	.26	.21	.08	.05	.03	.97	.89	.74	.59	.22	.13	.06	.97	.99	.96	.85	.27	.14	.06
	50, 7, 1			.98	.94	.43	.23	.10				.97	.45	.24	.10				.98	.45	.23	.09
	50, 7, 5		.99	.54	.40	.18	.10	.06		.97	.90	.80	.37	.21	.09	.95	.99	.98	.95	.40	.21	.07
	250, 3, 1			.99	.98	.42	.16	.03				.99	.47	.20	.04					.49	.22	.05
	250, 3, 5		.95	.09	.14	.08	.04	.02			.95	.87	.36	.16	.04					.47	.21	.05
	250, 5, 1					.82	.42	.06					.82	.42	.06					.82	.42	.05
	250, 5, 5			.56	.24	.13	.06	.02				.99	.72	.37	.06					.78	.39	.05
	250, 7, 1					.95	.60	.07					.94	.60	.07					.95	.60	.06
	250, 7, 5			.92	.66	.40	.22	.04					.89	.54	.07					.93	.55	.06
	500, 3, 1					.76	.35	.04					.78	.37	.05					.79	.38	.05
	500, 3, 5			.09	.20	.20	.08	.02				.99	.67	.31	.04					.76	.36	.05
	500, 5, 1					.98	.68	.06					.98	.68	.05					.98	.68	.05
	500, 5, 5			.79	.23	.14	.07	.03					.94	.61	.05					.97	.64	.05
	500, 7, 1						.85	.06						.85	.06						.85	.06
	500, 7, 5			.99	.79	.48	.30	.04					.99	.79	.06						.82	.05
0.5	50, 3, 1	.99	.92	.64	.40	.12	.08	.06		.97	.73	.47	.14	.09	.06			.83	.54	.15	.09	.06
	50, 3, 5	.94	.39	.12	.10	.04	.04	.03	.90	.71	.41	.27	.10	.07	.05		.98	.81	.53	.15	.10	.06
	50, 5, 1		.99	.93	.80	.29	.17	.10			.98	.84	.31	.19	.11			.99	.87	.32	.19	.11
	50, 5, 5		.89	.35	.28	.11	.07	.05	.98	.93	.78	.63	.26	.16	.10	.98	.99	.97	.84	.30	.17	.09
	50, 7, 1			.99	.95	.45	.27	.14				.96	.46	.27	.14				.97	.45	.27	.13
	50, 7, 5		.99	.64	.51	.23	.14	.09		.98	.93	.83	.40	.25	.13	.97		.99	.95	.42	.24	.10
	250, 3, 1				.98	.45	.21	.06				.99	.48	.23	.07					.49	.24	.08
	250, 3, 5		.93	.17	.14	.06	.03	.02			.97	.89	.38	.19	.06				.99	.46	.23	.08
	250, 5, 1					.79	.43	.09					.79	.44	.09					.79	.44	.09
	250, 5, 5			.68	.45	.23	.11	.03					.72	.39	.09					.76	.40	.08
	250, 7, 1					.92	.59	.11					.92	.59	.11					.93	.59	.10
	250, 7, 5			.95	.83	.59	.34	.07					.88	.55	.10					.91	.55	.09
	500, 3, 1					.74	.38	.07					.75	.38	.08					.76	.39	.08
	500, 3, 5			.20	.14	.10	.05	.02					.67	.34	.07					.74	.37	.07
	500, 5, 1					.96	.66	.09					.96	.66	.09					.96	.66	.09
	500, 5, 5			.86	.53	.31	.16	.03					.94	.61	.08					.96	.63	.08
	500, 7, 1						.83	.10						.82	.10						.83	.09
	500, 7, 5			.99	.93	.77	.53	.07					.99	.78	.09						.80	.08

See Table 8

**Table 11:** Monte Carlo results for $\alpha =-0.5$. $\varvec{\varUpsilon }^{(4),(6)}$

$\theta$	$\{N,T,\tau \}$ $\backslash \lambda$	$\varvec{\varUpsilon }^{(4)}$							$\varvec{\varUpsilon }^{(5)}$						
		$-$.70	$-$.30	$-$.10	$-$.05	$-$.01	$-$.005	.00	$-$.70	$-$.30	$-$.10	$-$.05	$-$.01	$-$.005	.00
0.0	50, 3, 1		.95	.74	.46	.11	.06	.04	.98	.92	.68	.43	.10	.06	.04
	50, 3, 5		.50	.45	.26	.09	.05	.04	.58	.30	.22	.17	.07	.04	.03
	50, 5, 1		.99	.98	.86	.30	.15	.07		.98	.95	.83	.28	.15	.07
	50, 5, 5		.68	.82	.63	.27	.14	.07	.92	.57	.44	.43	.19	.11	.05
	50, 7, 1				.97	.47	.25	.10			.99	.95	.45	.24	.10
	50, 7, 5		.84	.94	.81	.46	.25	.10		.82	.62	.62	.34	.20	.08
	250, 3, 1				.99	.50	.21	.04				.99	.47	.20	.04
	250, 3, 5		.95	.98	.89	.47	.20	.04	.91	.59	.58	.60	.28	.13	.03
	250, 5, 1					.84	.44	.06					.82	.42	.05
	250, 5, 5		.97		.99	.84	.47	.06		.89	.81	.88	.67	.35	.05
	250, 7, 1					.96	.63	.07					.94	.60	.07
	250, 7, 5		.99			.95	.66	.07		.99	.92	.96	.87	.54	.06
	500, 3, 1					.81	.39	.05					.78	.37	.05
	500, 3, 5		.99		.99	.80	.39	.04	.98	.78	.81	.85	.57	.27	.03
	500, 5, 1					.98	.71	.05					.98	.68	.05
	500, 5, 5					.99	.73	.05		.97	.94	.98	.93	.60	.05
	500, 7, 1						.87	.06						.85	.06
	500, 7, 5						.88	.06			.98		.99	.80	.06
0.5	50, 3, 1		.96	.74	.49	.14	.10	.07	.99	.94	.70	.46	.14	.09	.06
	50, 3, 5		.58	.48	.30	.11	.08	.05	.68	.39	.28	.21	.09	.06	.04
	50, 5, 1			.98	.85	.33	.19	.11		.99	.96	.83	.31	.19	.11
	50, 5, 5		.76	.85	.66	.30	.18	.10	.95	.67	.54	.50	.23	.15	.08
	50, 7, 1				.96	.47	.28	.14			.99	.95	.46	.27	.14
	50, 7, 5		.90	.96	.84	.47	.28	.14		.88	.71	.69	.37	.23	.12
	250, 3, 1				.99	.50	.24	.07				.99	.48	.23	.07
	250, 3, 5		.97	.99	.91	.47	.23	.06	.96	.78	.73	.71	.33	.17	.05
	250, 5, 1					.81	.45	.10					.79	.44	.09
	250, 5, 5		.99		.99	.81	.46	.09		.95	.92	.95	.70	.38	.09
	250, 7, 1					.93	.60	.10					.92	.59	.11
	250, 7, 5					.94	.63	.10			.97	.99	.87	.55	.10
	500, 3, 1					.77	.41	.08					.75	.39	.08
	500, 3, 5					.77	.40	.07	.99	.93	.92	.93	.62	.31	.06
	500, 5, 1					.97	.68	.09					.96	.66	.09
	500, 5, 5					.97	.70	.08		.99	.98	.99	.93	.61	.08
	500, 7, 1						.84	.10						.83	.10
	500, 7, 5						.85	.09					.99	.79	.09

See Table 8

## Footnotes

1
$\rho (\varvec{A})\equiv \max _{k}(|\lambda _{k}|)$, where $\lambda _{k}$ are (possibly complex) eigenvalues of a matrix $\varvec{A}$.

2
$$\ddot{\varvec{y}}_{i}\equiv \bar{\varvec{y}}_{i}-\varvec{y}_{i,0}$$ and $$\ddot{\varvec{y}}_{i-}\equiv \bar{\varvec{y}}_{i-}-\varvec{y}_{i,0}$$.

3 Unlike time series models, we do not define cointegration as a property of time series, as in our setup we keep *T* fixed.

4 We slightly abuse the notation in this case, so that it remains consistent with the general practice of the time series cointegration literature.

5
Dhaene and Jochmans (2016) prove singularity of the hessian matrix for $m=1$.

6 However, in this setup we still, for simplicity, assume that the initial observation has a zero mean, i.e. ${\text {E}}[\Delta {}\varvec{y}_{i,1}]=\varvec{0}_{m}$.

7 Here subscript *r* is introduced to highlight that these matrices are of rank *r*.

8
Kruiniger (2013) extended their results by allowing cross-sectional heteroscedasticity in the error terms.

9 Our numerical simulations suggest that the rank of ${\varvec{\mathcal {H}}}_{\ell }$ has rank deficiency larger than one (for $m=2$ the rank of ${\varvec{\mathcal {H}}}_{\ell }$ is equal to 7, while full rank is 10), hence results of Roznitzky et al. (2000) need to be generalized taking into account this possibility.

10 In particular, setups of BHP were considered.

11 See e.g. Magnus and Neudecker (2007).

12 Note that for large *T* consistent estimation is possible, but with non-standard distribution theory, see Phillips (2015).

13 Also referred as “mean non-stationarity”, see e.g. Bun and Sarafidis (2015).

14 Here $\varvec{C}\equiv \varvec{\beta }_{\perp }\left( \varvec{\alpha }_{\perp }'\varvec{\beta }_{\perp }\right) ^{-1}\varvec{\alpha }_{\perp }'$ is an $m-r$ rank matrix, while $\alpha _{\perp }, \varvec{\beta }_{\perp }$ are the orthogonal complements of $\alpha , \varvec{\beta }$.

15 In (DGP.3) for $\rho (\varvec{\varPhi })<1$ we have $\varvec{C}=\mathbf {O}_{m}$, resulting in stationary initialization. On the other hand, $\varvec{\varPhi }=\varvec{I}_{m}$ implies $\varvec{C}=\varvec{I}_{m}$ (by definition) so that (DGP.3) and (DGP.2) coincide (by redefining *M* to $M+1$).

16
$\overline{\Delta \varvec{y}_{i,t}\varvec{y}_{i,t-1}'}$ satisfies **Assumption 1** (asymptotic normal distribution), while $\varvec{V}$ satisfies **Assumption 2**(full rank). Note that if $\varvec{V}$ is of reduced rank the result in Theorem 2 can be modified as in Andrews (1987).

17 Note that positive definiteness of ${\text {E}}[\varvec{u}_{i,t-1}\varvec{y}_{i,t-1}']$ is a sufficient, but not a necessary condition. The term can be negative definite or even indefinite, as long as it has full rank.

18 In principle, other pooling schemes with weighted averages are possible, but for ease of exposition in this paper we consider simple time average.

19 However, in order to accommodate $\varvec{\eta }_{i}$ that does not satisfy the common dynamics assumption the data generating process of the initial condition needs to be modified.

20 For example if ${\text {var}}{\varepsilon _{i,0}}=\sigma ^{2}/(1-\phi ^{2})$.

21 As e.g. in Andrews and Cheng (2012).

22 For example in the panel unit root testing literature this issue is prominent, see discussion in Moon et al. (2007) and also Westerlund (2016).

23 Other studies, like Mutl (2009) adapted setups of BHP.

24 Results for $M=5$ are qualitatively and quantitatively similar to the ones presented in this paper.

25 Later we use notation $\varvec{\varUpsilon }^{(q)}$ with *q* indicating the particular element of this set.

26 Setting $S=50$ would be another option, but it is of similar arbitrariness.

27 As in this case orders of magnitude for *N* and *T* are not substantially different we suspect that critical values obtained as $N,T\rightarrow \infty$ (jointly) might be more appropriate.

28 Some preliminary MC results, not presented in this paper suggest that effect of $\tau$ in this setup is not-monotonic. In the sense that higher values of $\tau$ lead to increase of power rather than further decrease. At least for this particular design it seems that $\tau =5$ represents the close to worst possible scenario as minimum is reached for $\tau \approx 6.2$.

29 From $\varvec{\varUpsilon }^{(1)}$ some non-monotonicities are inherited. Apart from that, the superior test power properties (as compared to the effect stationary case) of $\varvec{\varUpsilon }^{(3)}$ are dominant. This combined behavior is due to the fact that $\varvec{\varUpsilon }^{(4)}$ is changing with $\lambda$. In designs with $\lambda$ substantially lower than 0 we have $\varvec{\varUpsilon }^{(4)}\approx \varvec{I}_{m}$, consecutively the *weak instrument problem under alternative* is less pronounced.

30 For a more detailed description of the data, please refer to Alonso-Borrego and Arellano (1999).

31 Other testing procedures are described below.

32 We focus only on testing $r=1$ vs. $r=2$, as e.g. using LR test based on the TML estimator the $H_{0}: r_{0}=0$ is rejected against the alternative $H_{A}: r_{0}=1$ with the value of the test statistic equal to 117.561. This value is substantial even if the $\chi ^{2}(1)$ does not provide a correct asymptotic approximation.

33 However, this testing procedure cannot be used if series are cointegrated.

34
Arellano (2003) presents some evidence of time-series heteroscedasticity in this dataset.
